# AMPK/PINK1/Parkin pathway-associated mitochondrial quality control contributes to magnesium sulfate-induced neuroprotection in postoperative cognitive dysfunction

**DOI:** 10.3389/fncel.2026.1969109

**Published:** 2026-10-28

**Authors:** Guangling Li, Bingqian Fan, Jing Tao, Yu Jiang, Yiwen Hua, Rui Zhan, Xianwen Hu

**Affiliations:** 1 Department of Anesthesiology, the Second Affiliated Hospital of Anhui Medical University, Hefei, Anhui, China; 2 Department of Anesthesiology, the Affiliated Hospital of Jiangnan University, Wuxi, Jiangsu, China; 3 Department of Anesthesiology, the First Affiliated Hospital of Bengbu Medical University, Bengbu, Anhui, China; 4 Department of Anesthesiology, Xuzhou Medical University, Xuzhou, Jiangsu, China; 5 Department of Anesthesiology, Xuancheng People’s Hospital, Xuancheng, Anhui, China

**Keywords:** AMPK/PINK1/Parkin signaling, magnesium sulfate, microglia, mitochondrial quality control, neuroinflammation, postoperative cognitive dysfunction

## Abstract

**Introduction:**

Postoperative cognitive dysfunction (POCD) is associated with neuroinflammation and mitochondrial dysregulation, but the mechanisms linking these processes remain incompletely defined. We investigated the neuroprotective effects of magnesium sulfate (MgSO₄) and the involvement of AMP-activated protein kinase (AMPK)/PTEN-induced kinase 1 (PINK1)/Parkin signaling in experimental POCD.

**Methods:**

POCD was induced in mice by open tibial fracture under isoflurane anesthesia, and an *in vitro* inflammatory model was established in lipopolysaccharide (LPS)-treated BV2 microglia. Behavioral tests, enzyme-linked immunosorbent assay, western blotting, TUNEL staining, JC-1 analysis, and adenosine triphosphate (ATP) measurement were used to assess cognition, inflammatory responses, mitochondrial-related metabolic alterations, and pathway-associated changes.

**Results:**

MgSO₄ improved postoperative behavioral deficits, reduced hippocampal inflammatory cytokines, normalized microglial activation-associated markers, improved cellular ATP levels and mitochondrial membrane potential alterations, and partially normalized mitochondrial dynamics-related protein changes. POCD or LPS reduced PINK1, Parkin, and LC3-II while increasing p62; MgSO₄ partially reversed these changes. MgSO₄ also restored p-AMPK levels, whereas the pharmacological AMPK inhibitor Compound C partially attenuated the effects of MgSO₄ on PINK1/Parkin signaling, ATP levels, inflammatory responses, mitochondrial markers, and behavioral outcomes.

**Discussion:**

These findings support a model in which AMPK/PINK1/Parkin-related mitochondrial quality control is involved in the neuroprotective effects of MgSO₄ in experimental POCD.

## 1 Introduction

Postoperative cognitive dysfunction (POCD) is a syndrome of cognitive impairment occurring after surgery and anesthesia, characterized by deficits in multiple cognitive domains including attention, memory, executive function, and processing speed. It can persist for weeks to months or longer, significantly impairing quality of life and increasing healthcare burdens. The incidence of POCD in older patients can reach 30%–40%, and persistent cognitive deficits may remain months after surgery (Zhang L. et al., 2024). Effective interventions that demonstrably improve long-term outcomes remain limited, underscoring the need to clarify the underlying mechanisms. Neuroinflammation, excessive microglial activation, and mitochondrial dysfunction have all been implicated in POCD. Surgical stress activates microglia in the hippocampus and increases inflammatory cytokine production, contributing to cognitive impairment (Niu et al., 2020). Emerging work further suggests that persistent changes in microglial inflammatory state and mitochondrial homeostasis may delay cognitive recovery (Wu et al., 2025). PINK1/Parkin-dependent mitophagy has been linked to the control of neuroinflammatory signaling, while pharmacological induction of mitophagy can improve neuroinflammation and cognitive dysfunction after surgery (Cao et al., 2021; Zhang et al., 2025b).

Mitochondrial quality control is essential for maintaining cellular energy homeostasis, adapting to oxidative stress, and limiting inflammatory signaling. AMP-activated protein kinase (AMPK), a key sensor of cellular energy status, participates in mitochondrial quality control by regulating metabolism, mitochondrial dynamics, and autophagy. PTEN-induced kinase 1 (PINK1)/Parkin-mediated mitophagy is a major pathway for the selective clearance of damaged mitochondria. Disruption of PINK1/Parkin signaling can lead to mitochondrial DNA accumulation and activation of inflammatory pathways, whereas enhancement of mitophagy may reduce damaged mitochondrial burden and inflammatory signaling (Zhang et al., 2025b; Jiang et al., 2024; Seabright and Lai, 2020). In microglia, mitochondrial stress can promote inflammatory activation, suggesting that the AMPK/PINK1/Parkin axis may provide a mechanistic link between mitochondrial quality control and neuroinflammation in POCD.

Perioperative pharmacological interventions are receiving increasing attention for the prevention of postoperative neurological complications. Magnesium sulfate (MgSO₄) is an established drug with a long clinical history and has been used in anesthesiology and neurology, including as an anticonvulsant in preeclampsia/eclampsia and as an adjunct to perioperative analgesia. MgSO₄ may reduce excitotoxicity through N-methyl-D-aspartate receptor modulation and can influence calcium signaling, inflammatory pathways, and neuronal energy metabolism (Jin and Zhao, 2025). *In vitro* studies have shown that MgSO₄ suppresses LPS-induced inflammatory mediator release from microglia (Gao et al., 2013), and animal studies suggest that it can modulate microglial inflammatory phenotypes and neuroinflammation (Wang Q. et al., 2025). MgSO₄ has also been reported to preserve cellular bioenergetic function and ATP levels under excitotoxic stress (Clerc et al., 2013) and to modify inflammatory and mitochondrial responses in models of cerebral injury (Le Dieu-Lugon et al., 2021). However, whether MgSO₄ coordinately regulates neuroinflammation and mitochondrial quality control in POCD, and whether AMPK/PINK1/Parkin signaling contributes to these effects, remains unclear. Therefore, this study investigated how MgSO₄ influences microglial inflammatory responses and mitochondrial quality control in complementary *in vivo* and *in vitro* models of POCD-related neuroinflammation, and examined the involvement of the AMPK/PINK1/Parkin pathway using the pharmacological AMPK inhibitor Compound C.

## 2 Materials and methods

### 2.1 Establishment of the POCD animal model and magnesium sulfate intervention

Adult male C57BL/6J mice aged 8–10 weeks and weighing 22–25 g were housed under controlled conditions at 22 °C ± 2 °C with a 12-h light/dark cycle and free access to food and water. The POCD model was established by open tibial fracture surgery under inhalational isoflurane anesthesia. Mice were anesthetized with 2% isoflurane (Sigma-Aldrich, St. Louis, MO, USA; Cat. No. 792632), and buprenorphine (0.1 mg/kg, subcutaneously) was administered after induction of anesthesia and before skin incision for perioperative analgesia.

After shaving and disinfecting the surgical area, a longitudinal skin incision was made over the right tibia under aseptic conditions. The subcutaneous tissue and surrounding soft tissue were carefully separated to expose the tibial shaft. The periosteum surrounding the planned fracture site was gently separated, and a standardized transverse osteotomy/fracture was created under direct visualization at the middle-to-distal portion of the tibia using sterile surgical instruments. After confirmation of the fracture, the surgical field was irrigated with sterile saline and the incision was closed layer by layer. Control animals underwent the corresponding isoflurane anesthesia and skin incision without fracture manipulation.

Magnesium sulfate (MgSO₄, anhydrous; Sigma-Aldrich, St. Louis, MO, USA; Cat. No. M7506) was dissolved in sterile saline and administered intraperitoneally at 50, 100, or 200 mg/kg once daily for three consecutive days, beginning 30 min before surgery. These doses corresponded to the low-, middle-, and high-dose MgSO₄ treatment conditions, respectively. Vehicle-treated mice received an equivalent volume of sterile vehicle. For subsequent mechanistic experiments, the MgSO₄ dose selected on the basis of the dose–response experiments was used. For AMPK inhibition, Compound C/dorsomorphin (MedChemExpress, Monmouth Junction, NJ, USA; Cat. No. HY-13418A) was dissolved in DMSO and diluted in sterile saline to a final DMSO concentration below 1%. Compound C was administered intraperitoneally at 20 mg/kg 30 min before MgSO₄ treatment.

Following surgery, mice were allowed to recover in a warmed environment and were returned to their home cages after regaining adequate spontaneous activity. Additional analgesic treatment was provided in accordance with the approved institutional animal-care protocol when indicated. Animals were monitored at least daily and more frequently during the immediate postoperative recovery period for general appearance, spontaneous activity, mobility, food and water intake, body weight, wound condition, and signs of pain or distress. Prespecified humane endpoints included severe or persistent impairment of mobility, inability to access food or water, marked body-weight loss, wound dehiscence or infection, or signs of severe unrelieved pain or distress. Animals reaching a humane endpoint were to be removed from the study and euthanized according to the approved institutional protocol; no animals reached the predefined humane endpoints during the study. The study was approved by the Institutional Animal Care and Use Committee of Anhui Medical University (Approval No. LLSC20241789), and all procedures were conducted in accordance with relevant institutional regulations and the ARRIVE guidelines.

### 2.2 LPS-induced BV2 microglial model and drug treatment

BV2 murine microglial cells were obtained from the Cell Bank of Type Culture Collection of the Chinese Academy of Sciences (Shanghai, China; Cat. No. SCSP-5208). Cells were maintained in high-glucose DMEM supplemented with 10% fetal bovine serum, 100 U/mL penicillin, and 100 μg/mL streptomycin at 37 °C in a humidified atmosphere containing 5% CO₂. Cells between passages 10 and 20 were used at approximately 70%–80% confluence. To establish an inflammatory activation model, BV2 cells were stimulated with lipopolysaccharide (LPS, 1 μg/mL) for 24 h (Li et al., 2025). Before LPS stimulation, BV2 cells were pretreated with MgSO₄ for 2 h at three concentrations (5, 10, and 20 mM), which were defined as low-, middle-, and high-dose conditions, respectively. The 10 mM MgSO₄ treatment condition was selected for subsequent western blotting and pathway-intervention experiments based on the dose–response analysis. For AMPK inhibition experiments, BV2 cells were pretreated with Compound C/dorsomorphin (10 μM) for 1 h before MgSO₄ treatment, followed by LPS stimulation for 24 h. Vehicle-treated controls were analyzed in parallel.

### 2.3 JC-1 mitochondrial membrane potential assay

Mitochondrial membrane potential (ΔΨm) in BV2 cells was assessed using a JC-1 mitochondrial membrane potential assay kit (Beyotime Biotechnology, Shanghai, China; Cat. No. C2006). After treatment, cells were incubated with JC-1 working solution according to the manufacturer’s instructions at 37 °C in the dark for 20–30 min and then washed with JC-1 buffer. JC-1 forms red fluorescent aggregates at relatively high mitochondrial membrane potential and remains as green fluorescent monomers when the membrane potential decreases. The red/green fluorescence signal was used to quantify relative changes in mitochondrial membrane potential.

### 2.4 TUNEL staining

Apoptotic cell death in BV2 microglia and hippocampal tissues was assessed using the *In Situ* Cell Death Detection Kit, TMR red (Roche/Sigma-Aldrich; Cat. No. 12156792910) according to the manufacturer’s instructions. For BV2 cells, cells were fixed with 4% paraformaldehyde for 30 min, permeabilized with 0.1% Triton X-100 in 0.1% sodium citrate for 2 min on ice, and incubated with the TUNEL reaction mixture at 37 °C for 60 min in the dark. After DAPI counterstaining, fluorescence images were captured by fluorescence microscopy. TUNEL-positive cells were quantified from at least five randomly selected fields per sample and expressed as relative values for between-group comparisons. For hippocampal sections, 20-μm frozen sections were permeabilized according to the manufacturer’s protocol and incubated with the TUNEL reaction mixture under the same conditions. After DAPI counterstaining, images were acquired using confocal microscopy.

### 2.5 ATP measurement

ATP levels in hippocampal tissue and BV2 cells were measured using an ATP Assay Kit (Beyotime Biotechnology, Shanghai, China; Cat. No. S0026) according to the manufacturer’s instructions. Tissue or cell samples were processed with the kit-provided ATP lysis buffer, and ATP-dependent chemiluminescence was quantified against the ATP standard supplied with the kit. Results are presented as relative ATP readouts for the corresponding experimental comparisons.

### 2.6 Behavioral assays and experimental timeline

Behavioral testing was performed during the early postoperative period by investigators blinded to group allocation. The first MgSO₄ dose was administered 30 min before surgery on day 0, followed by the second and third doses on postoperative days 1 and 2 (POD1 and POD2), respectively. To minimize interference between behavioral paradigms, spatial learning and memory, locomotor activity, anxiety-like behavior, and recognition memory were assessed according to a predefined postoperative schedule.

#### 2.6.1 Morris water maze

Spatial learning was assessed using the Morris water maze (MWM) beginning on POD1. Mice underwent acquisition training daily from POD1 to POD5. During each training session, mice were released into the pool from predefined starting positions and allowed to locate the submerged escape platform. Escape latency was recorded automatically using a video-tracking system. Swimming speed during the acquisition phase was simultaneously recorded and analyzed to evaluate whether postoperative motor impairment affected Morris water maze performance. The escape-latency values obtained from POD1 to POD5 were used to construct the postoperative learning curves. A probe trial was performed on POD6, 24 h after completion of the final acquisition session. The platform was removed, mice were allowed to swim freely, and the prespecified latency and error measures were recorded as indices of spatial memory performance.

#### 2.6.2 Open-field test

Spontaneous locomotor activity and anxiety-like behavior were assessed using the open-field test (OFT) on POD7. Each mouse was placed individually in the center of the open-field arena and allowed to explore freely under quiet and constant lighting conditions. Total distance traveled and the number of entries into the central zone were automatically recorded using a video-tracking system. The apparatus was cleaned between animals to minimize olfactory cues.

#### 2.6.3 Novel object recognition test

Recognition memory was assessed using the novel object recognition (NOR) test after the OFT. Habituation was performed on POD7, familiarization on POD8, and the recognition test on POD9. During familiarization, mice were allowed to explore two identical objects. During the test phase, one familiar object was replaced with a novel object. Exploration times for the novel and familiar objects were recorded automatically, and the discrimination rate was calculated from the relative exploration of the two objects.

#### 2.6.4 Tissue collection

A separate cohort of mice assigned to early molecular analyses was euthanized on POD3, after completion of the three-dose perioperative MgSO₄ regimen. Mice were deeply anesthetized with isoflurane and, after confirmation of a deep plane of anesthesia, euthanasia was completed by cervical dislocation in accordance with the approved institutional animal-care protocol. Death was confirmed before tissue collection. The brain was rapidly removed, and bilateral hippocampi were dissected on ice and either processed immediately or stored at −80 °C for subsequent ELISA, ATP measurement, and western blot analyses. A separate behavioral cohort underwent the complete postoperative behavioral-testing sequence and was euthanized after completion of the final behavioral assessment on POD9. Hippocampal tissue was collected from these animals when required for corresponding endpoint analyses.

### 2.7 Enzyme-linked immunosorbent assay

Inflammatory cytokines were quantified by enzyme-linked immunosorbent assay (ELISA). For hippocampal measurements, bilateral hippocampal tissue was harvested after euthanasia under deep anesthesia, homogenized on ice in pre-chilled buffer, and centrifuged at 4 °C to collect the supernatant. TNF-α, IL-1β, and IL-6 were measured using the Mouse TNF-α ELISA Kit (Beyotime Biotechnology, Shanghai, China; Cat. No. PT512), Mouse IL-1β ELISA Kit (Beyotime Biotechnology; Cat. No. PI301), and Mouse IL-6 ELISA Kit (Beyotime Biotechnology; Cat. No. PI326), respectively, according to the manufacturer’s instructions. The same kits were used to quantify the corresponding cytokines in BV2 cell-culture supernatants. Absorbance was read at 450 nm and cytokine concentrations were calculated from the corresponding standard curves.

### 2.8 Isolation of hippocampal microglia

To further evaluate whether surgery-associated AMPK/PINK1/Parkin pathway alterations were present in hippocampal microglia, microglial cells were isolated from freshly collected hippocampal tissues for subsequent western blot and ELISA analyses. Briefly, bilateral hippocampi were rapidly dissected after tissue collection and processed into single-cell suspensions using mechanical dissociation and enzymatic digestion according to the manufacturer’s instructions. The cell suspension was filtered to remove tissue debris and subjected to CD11b-based magnetic-activated cell sorting to obtain hippocampal microglial fractions. The isolated microglial fractions were processed separately for western blot and ELISA analyses. For western blotting, protein lysates from the isolated hippocampal microglia were prepared and analyzed as described in Section 2.9. PINK1, Parkin, p62, and p-AMPK were examined by immunoblotting, with β-actin used as the loading control. For ELISA, isolated microglial fractions were lysed, and p-AMPK, PINK1, Parkin, and p62 were quantified using ELISA kits according to the manufacturers’ protocols.

### 2.9 Western blot analysis

Western blotting was used to determine the expression of proteins associated with microglial activation, apoptosis, mitochondrial quality control, mitophagy-related signaling, and AMPK signaling, including the mitochondrial dynamics-related proteins DRP1 and OPA1, as well as the mitochondrial marker TOMM20. Hippocampal tissue, isolated hippocampal microglia, and treated BV2 cells were lysed in RIPA buffer containing protease and phosphatase inhibitors and clarified by centrifugation at 4 °C. Protein concentrations were determined using the BCA method. Equal amounts of protein were separated by SDS-PAGE and transferred onto PVDF membranes. After blocking with 5% skim milk or bovine serum albumin for 1 h at room temperature, membranes were incubated overnight at 4 °C with primary antibodies against PINK1 (Proteintech, Rosemont, IL, USA; Cat. No. 23274-1-AP; 1:1,000), Parkin/PARK2 (Proteintech; Cat. No. 14060-1-AP; 1:1,000), AMPKα (Immunoway; Cat. YT0216; 1:1,000), phospho-AMPKα (Thr172) (Immunoway; Cat. YP1259; 1:1,000), CD68 (Proteintech; Cat. No. 28058-1-AP; 1:1,000), Iba1/AIF1 (Proteintech; Cat. No. 10904-1-AP; 1:1,000), CD206/MRC1 (Proteintech; Cat. No. 18704-1-AP; 1:1,000), Bax (Proteintech; Cat. No. 50599-2-Ig; 1:2,000), Bcl-2 (Proteintech; Cat. No. 68103-1-Ig; 1:2,000), DRP1/DNM1L (Proteintech; Cat. No. 12957-1-AP; 1:1,000), OPA1 (Proteintech; Cat. No. 27733-1-AP; 1:1,000), TOMM20 (Proteintech; Cat. No. 11802-1-AP; 1:1,000), LC3B (Proteintech; Cat. No. 18725-1-AP; 1:1,000), p62/SQSTM1 (Proteintech; Cat. No. 18420-1-AP; 1:1,000), and β-actin (Proteintech; Cat. No. 66009-1-Ig; 1:5,000). After washing with TBST, membranes were incubated for 1 h at room temperature with HRP-conjugated Goat Anti-Mouse IgG (H + L) (Proteintech; Cat. No. SA00001-1; 1:5,000) or HRP-conjugated Goat Anti-Rabbit IgG (H + L) (Proteintech; Cat. No. SA00001-2; 1:5,000), as appropriate. Protein bands were visualized using enhanced chemiluminescence and quantified using ImageJ software. The expression levels of target proteins were normalized to β-actin, which was used as the loading control. Quantification was performed from independent biological replicates, and relative protein expression was calculated as the ratio of target protein intensity to β-actin intensity. All western blot experiments were performed using at least three independent biological replicates. The quantitative analysis corresponding to the WB bands are shown in Figures S2-S6.

### 2.10 Histology and immunofluorescence staining

Mice were deeply anesthetized and transcardially perfused with ice-cold phosphate-buffered saline (PBS), followed by fixation with 4% paraformaldehyde (PFA) in PBS. Brains were removed, post-fixed in 4% PFA overnight at 4 °C, dehydrated in graded sucrose solutions, embedded in optimal cutting temperature (OCT) compound, and coronally sectioned at 20 μm thickness using a cryostat. For histological evaluation, hippocampal sections were subjected to hematoxylin and eosin (H&E) staining and Nissl staining according to standard protocols. Neuronal morphology, cellular organization, and neuronal integrity in hippocampal regions were examined by light microscopy.

For immunofluorescence staining, hippocampal sections were blocked with 5% bovine serum albumin (BSA) containing 0.3% Triton X-100 for 1 h at room temperature and incubated overnight at 4 °C with primary antibodies against neuronal marker NeuN (1:500, Abcam, ab177487), microglial marker Iba1 (1:500, Wako, 019-19741), activated microglial marker CD68 (1:200, Abcam, ab53444), mitochondrial marker TOMM20 (1:500, Proteintech, 11802-1-AP), and lysosomal marker LAMP1 (1:500, Abcam, ab24170). After washing with PBS, sections were incubated with appropriate Alexa Fluor-conjugated secondary antibodies (1:500, Invitrogen) for 1 h at room temperature in the dark. Nuclei were counterstained with DAPI (1:1,000, Sigma-Aldrich).

For assessment of mitochondrial–lysosomal colocalization within microglia, hippocampal sections were subjected to triple immunofluorescence staining for Iba1, TOM20, and LAMP1. Colocalization signals between TOM20 and LAMP1 within Iba1-positive microglial cells were analyzed using confocal microscopy.

### 2.11 Statistical analysis

Statistical analyses were performed using GraphPad Prism 10.0 (GraphPad Software, San Diego, CA, United States). Data are presented as mean ± standard deviation (SD). For animal experiments, n represents the number of independent animals. For *in vitro* experiments, n represents independent biological replicates from separate experiments. For comparisons among three or more independent groups at a single time point, one-way analysis of variance (ANOVA) followed by Tukey’s multiple-comparisons test was used. Escape-latency data collected across consecutive Morris water-maze training days were analyzed using two-way repeated-measures ANOVA, with treatment group as the between-subject factor and training day as the within-subject factor, followed by Šídák’s multiple-comparisons test where appropriate. All tests were two-sided, and *p* < 0.05 was considered statistically significant. Individual observations are displayed where applicable. Unless otherwise specified, n represents independent biological samples or animals rather than technical replicates. Sample sizes are provided in the corresponding figure legends. Statistical significance is indicated as *p < 0.05, **p < 0.01, ***p < 0.001, and ****p < 0.0001; ns indicates no statistically significant difference.

## 3 Results

### 3.1 MgSO₄ attenuates hippocampal neuroinflammation and mitochondrial quality-control abnormalities after surgery

Surgery markedly increased hippocampal TNF-α, IL-1β, and IL-6 levels, whereas MgSO₄ progressively reduced these inflammatory responses across the treatment groups (Figure 1C). Western blotting showed that surgery reduced CD206 and Bcl-2 while increasing CD68, Iba1, and Bax. MgSO₄ partially reversed these surgery-associated alterations (Figures 1A,B). Surgery was also associated with altered mitochondrial homeostasis, characterized by increased DRP1 and reduced OPA1 and TOMM20. MgSO₄ partially normalized these mitochondrial markers (Figures 1D,F). In parallel, surgery reduced PINK1, Parkin, and LC3-II and increased p62, whereas MgSO₄ partially reversed this protein-expression pattern (Figures 1D,E). Together, these findings indicate that MgSO₄ attenuates postoperative hippocampal neuroinflammation and is associated with restoration of mitochondrial quality-control-related protein profiles. Furthermore, triple immunofluorescence staining for Iba1, TOMM20, and LAMP1 demonstrated decreased mitochondrial–lysosomal colocalization within hippocampal microglia after surgery, whereas MgSO₄ partially restored TOMM20-LAMP1 overlap, supporting recovery of mitochondrial quality-control-associated processes (Figure 1G).

**Figure 1:**
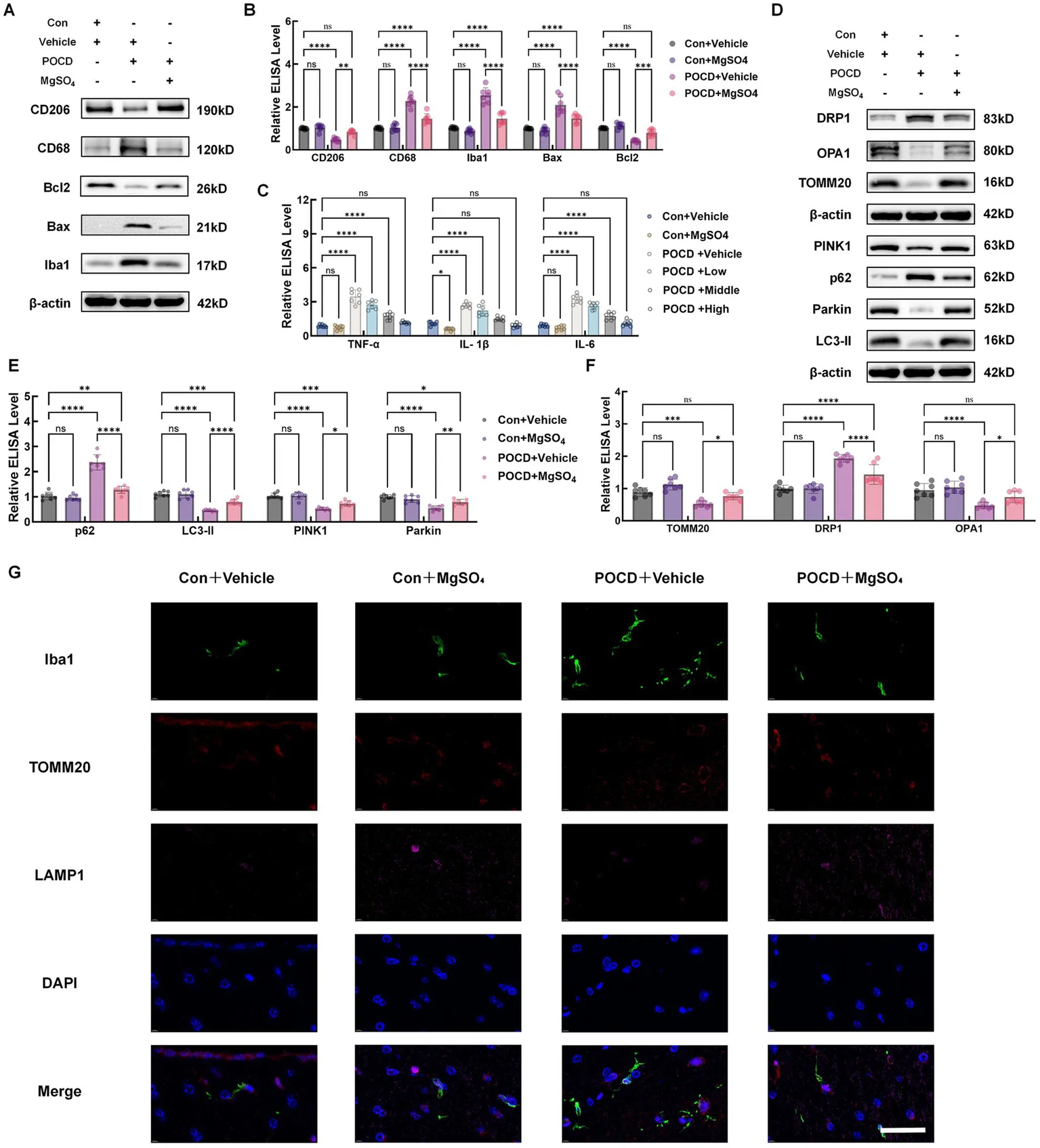
MgSO_4_ attenuates postoperative hippocampal neuroinflammation and mitochondrial quality-control abnormalities. **(A)** Representative western blots of CD206, CD68, Bcl-2, Bax, and Iba1 in hippocampal tissue. **(B)** Relative levels of CD206, CD68, Iba1, Bax, and Bcl-2 determined by ELISA. **(C)** ELISA measurement of TNF-α, IL-1β, and IL-6 in hippocampal tissue (*n* = 6 mice per group). **(D)** Representative western blots of DRP1, OPA1, TOMM20, PINK1, p62, Parkin, and LC3-II in hippocampal tissue. **(E)** Relative ELISA levels of p62, LC3-II, PINK1, and Parkin (*n* = 7 biological samples per group). **(F)** Relative ELISA levels of TOMM20, DRP1, and OPA1 (*n* = 7 biological samples per group). **(G)** Triple immunofluorescence staining for Iba1, TOMM20, and LAMP1 in hippocampal tissue. Western blotting was performed using at least three independent biological replicates per group, and β-actin served as the loading control. Data are presented as mean ± SD. Statistical significance was determined using one-way ANOVA followed by Tukey’s multiple comparisons test for comparisons among multiple groups. Behavioral data obtained from repeated measurements were analyzed using two-way repeated-measures ANOVA followed by Šídák’s multiple comparisons test. *p < 0.05, **p < 0.01, ***p < 0.001, ****p < 0.0001; ns, not significant.

Histological evaluation of hippocampal neuronal integrity revealed substantial neuronal injury after surgery and the neuroprotective effects of MgSO₄. HE staining demonstrated increased structural disruption, whereas Nissl staining showed reduced neuronal integrity in the hippocampus of POCD mice. NeuN immunofluorescence further confirmed a reduction in surviving hippocampal neurons, and TUNEL staining revealed increased apoptotic cell death. MgSO₄ administration significantly alleviated these pathological changes, preserving neuronal morphology, increasing NeuN-positive neurons, and reducing TUNEL-positive cells compared with POCD+Vehicle mice. Immunofluorescence staining showed that Iba1-positive microglial accumulation and CD68 expression are increased in the hippocampus of POCD group, accompanied by activated microglial morphological changes. MgSO₄ treatment reduced microglial activation-associated alterations (Figure 2).

**Figure 2:**
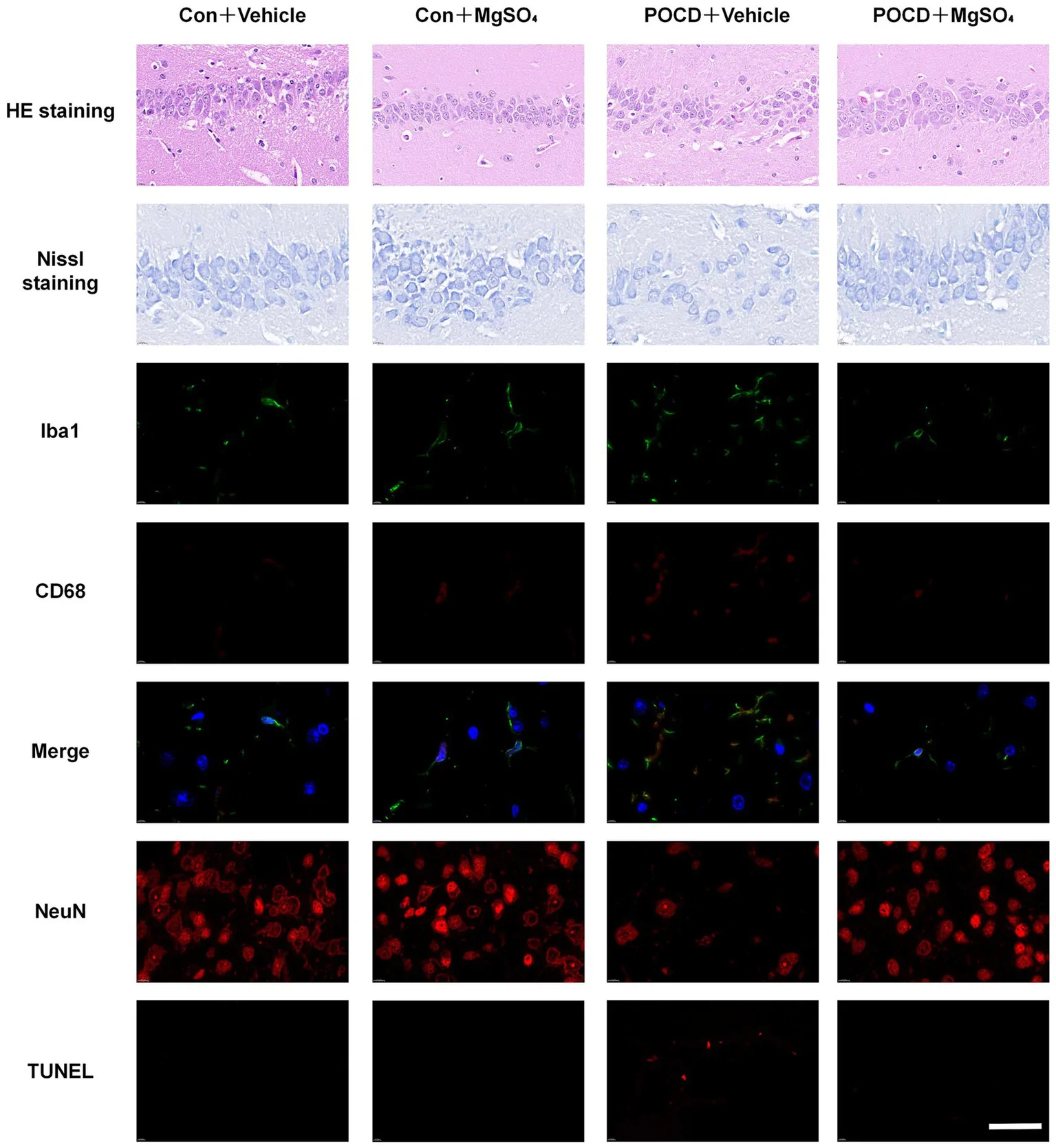
Hippocampal histological assessment following surgery and MgSO₄ treatment. Representative H&E staining, Nissl staining, Iba1 and CD68 immunofluorescence, NeuN immunofluorescence, TUNEL staining. Scale bar = 100 μm.

### 3.2 MgSO₄ restores p-AMPK and ATP levels and alleviates neurobehavioral deficits

Compared with the control group, postoperative mice showed markedly reduced hippocampal p-AMPK, whereas MgSO₄ restored p-AMPK levels (Figures 3A,B). Hippocampal ATP levels were also reduced after surgery and showed a dose-related recovery following MgSO₄ treatment (Figure 3C). In the Morris water maze, surgery increased escape latency and the number of errors, whereas MgSO₄ improved both measures (Figure 3D). Consistent with these findings, postoperative escape-latency curves showed impaired acquisition after surgery and improvement following MgSO₄ treatment (Figure 3E). No significant differences were found in swimming speed during MWM acquisition between POD1 to POD5, indicating that differences in escape latency were unlikely to be primarily attributable to altered swimming ability (Supplementary Figure S1). In the novel object recognition test, surgery reduced exploration time and the discrimination rate, both of which were improved by MgSO₄ (Figure 3F). Surgery also reduced total distance traveled and entries into the central zone in the open-field test, whereas MgSO₄ partially restored these behavioral measures (Figure 3G).

**Figure 3:**
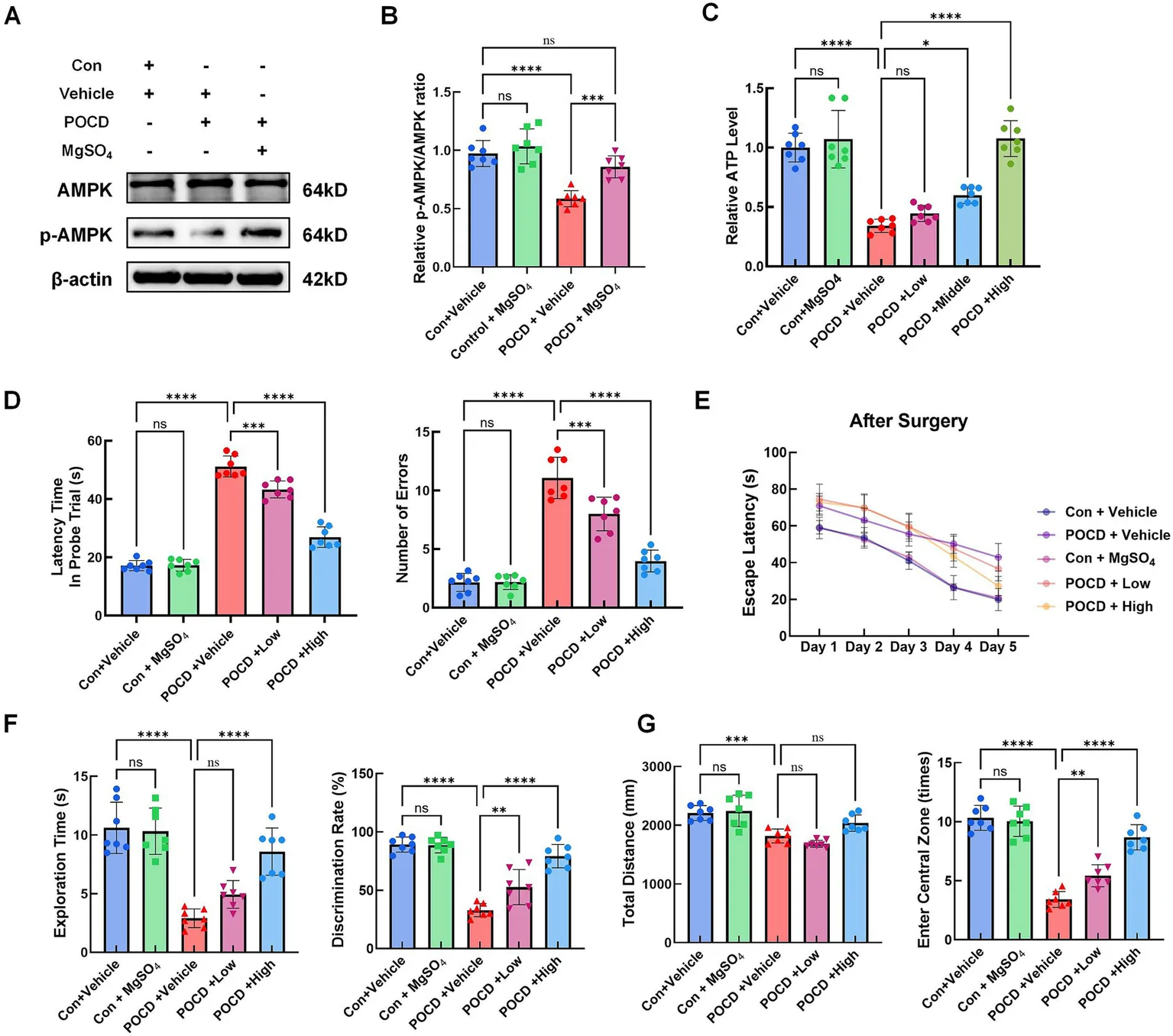
MgSO_4_ restores p-AMPK and ATP levels and alleviates neurobehavioral deficits in postoperative mice. **(A)** Representative western blots of total AMPK and p-AMPK in hippocampal tissue. **(B)** ELISA analysis of p-AMPK (*n* = 7 biological samples per group). **(C)** Relative hippocampal ATP levels (*n* = 6 mice per group). **(D)** MWM probe-trial latency and error measures on POD6 (*n* = 6 mice per group). **(E)** Escape-latency learning curves during MWM acquisition from POD1 to POD5 (*n* = 6 mice per group). **(F)** Exploration time and discrimination rate in the NOR test on POD9 (*n* = 6 mice per group). **(G)** Total distance traveled and central-zone entries in the OFT on POD7 (*n* = 6 mice per group). Data are presented as mean ± SD. Statistical significance was determined using one-way ANOVA followed by Tukey’s multiple comparisons test for comparisons among multiple groups. Behavioral data obtained from repeated measurements were analyzed using two-way repeated-measures ANOVA followed by Šídák’s multiple comparisons test. *p < 0.05, **p < 0.01, ***p < 0.001, ****p < 0.0001; ns, not significant. Swimming speed data during MWM acquisition are shown in Supplementary Figure S1.

### 3.3 MgSO₄ attenuates LPS-induced inflammatory activation and mitochondrial stress-associated alterations in BV2 microglia

In LPS-treated BV2 microglia, CD206 and Bcl-2 were reduced, whereas CD68, Iba1, and Bax were increased. MgSO₄ partially reversed these LPS-associated changes (Figures 4A,B). LPS also markedly reduced p-AMPK, whereas MgSO₄ restored p-AMPK levels (Figures 4C,D). In dose–response experiments, LPS increased TNF-α, IL-1β, and IL-6 levels, whereas increasing concentrations of MgSO₄ (5, 10, and 20 mM) progressively attenuated these inflammatory responses (Figure 4E).

**Figure 4:**
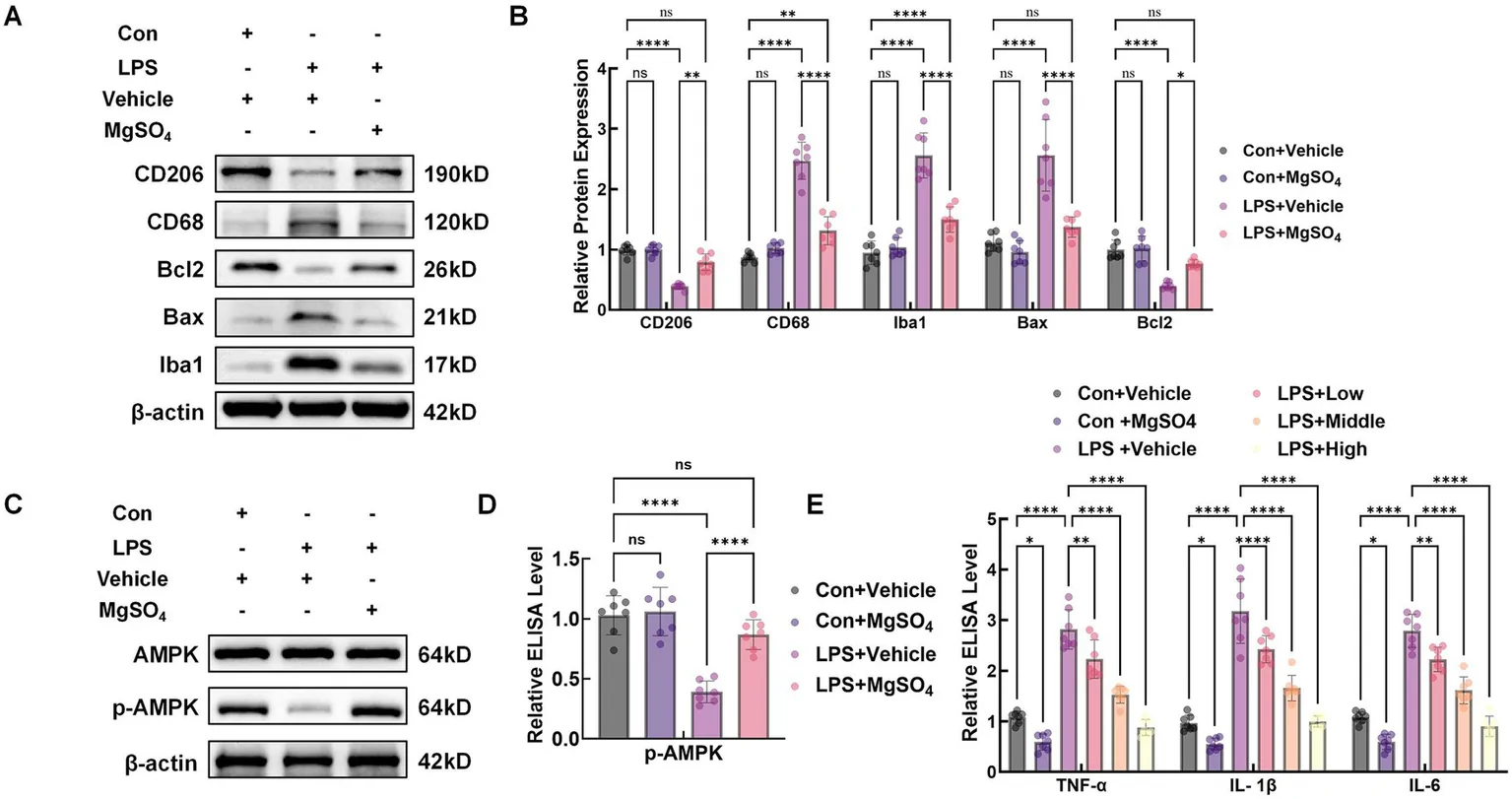
MgSO_4_ attenuates LPS-induced inflammatory activation and restores p-AMPK in BV2 microglia. **(A,B)** Representative western blots and ELISA analysis of CD206, CD68, Iba1, Bax, and Bcl-2. Densitometric values were normalized to β-actin (*n* = 3 independent biological samples per group). **(C,D)** Representative western blots of total AMPK and p-AMPK and ELISA analysis of p-AMPK (*n* = 7 independent biological samples per group). **(E)** ELISA measurement of TNF-α, IL-1β, and IL-6 under control, LPS, and increasing MgSO_4_ treatment conditions (*n* = 6 per group). Data are presented as mean ± SD. Statistical significance was determined using one-way ANOVA followed by Tukey’s multiple comparisons test for comparisons among multiple groups. Behavioral data obtained from repeated measurements were analyzed using two-way repeated-measures ANOVA followed by Šídák’s multiple comparisons test. *p < 0.05, **p < 0.01, ***p < 0.001, ****p < 0.0001; ns, not significant.

TUNEL analysis showed a marked increase in relative TUNEL-positive cells after LPS stimulation, which was progressively reduced by MgSO₄ treatment (Figure 5A). LPS also reduced relative ATP levels and mitochondrial membrane potential, whereas MgSO₄ partially improved these mitochondrial-related metabolic readouts (Figures 5B,C). To determine whether surgery-associated alterations in AMPK/PINK1/Parkin occurred specifically in hippocampal microglia, CD11b-positive microglia were isolated from hippocampal tissues using magnetic-activated cell sorting. Western blotting was performed on the isolated hippocampal microglia to examine PINK1, p62, Parkin, and p-AMPK (Figure 5D). ELISA analysis of the isolated hippocampal microglia showed that surgery significantly reduced p-AMPK, PINK1, and Parkin levels and increased p62, whereas MgSO₄ treatment partially restored these changes (Figure 5E).

**Figure 5:**
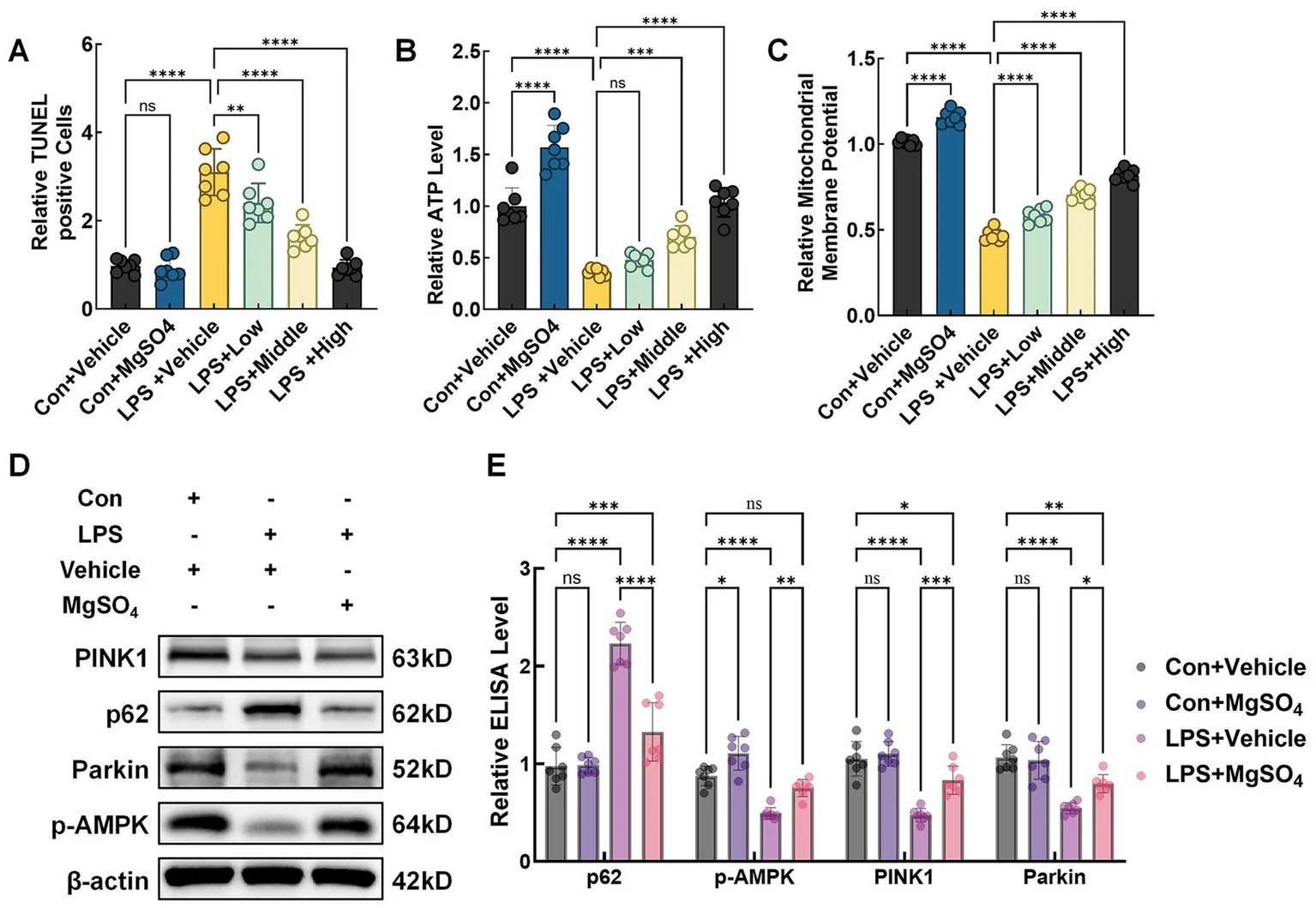
MgSO_4_ attenuates mitochondrial stress-associated alterations in BV2 microglia and is associated with mitochondrial quality-control-related protein changes in isolated hippocampal microglia. **(A)** Quantification of relative TUNEL-positive BV2 microglia cells (*n* = 6 per group). **(B)** Relative ATP readouts of BV2 microglia cells (*n* = 6 per group). **(C)** Relative mitochondrial membrane potential assessed by JC-1 in BV2 cells (*n* = 6 per group). **(D)** Representative western blots of PINK1, p62, Parkin, and p-AMPK in microglia isolated from mouse hippocampal tissue. **(E)** ELISA analysis of p62, p-AMPK, PINK1, and Parkin in microglia isolated from mouse hippocampal tissue (*n* = 7 biological samples per group). Data are presented as mean ± SD. Statistical significance was determined using one-way ANOVA followed by Tukey’s multiple comparisons test for comparisons among multiple groups. Behavioral data obtained from repeated measurements were analyzed using two-way repeated-measures ANOVA followed by Šídák’s multiple comparisons test. *p < 0.05, **p < 0.01, ***p < 0.001, ****p < 0.0001; ns, not significant.

### 3.4 Compound C partially attenuates the protective effects of MgSO₄ in LPS-treated BV2 microglia

To examine the involvement of AMPK signaling, Compound C was introduced as a pharmacological AMPK inhibitor. LPS reduced CD206 and increased CD68 and Iba1, whereas MgSO₄ partially normalized these microglial activation-associated markers. Compound C weakened the effects of MgSO₄ (Figures 6A,B). MgSO₄ also attenuated the LPS-associated increases in TNF-α, IL-1β, and IL-6, whereas Compound C partially diminished this anti-inflammatory effect (Figure 6C). MgSO₄ restored relative ATP levels after LPS stimulation, and this restoration was attenuated in the presence of Compound C (Figure 6D).

**Figure 6:**
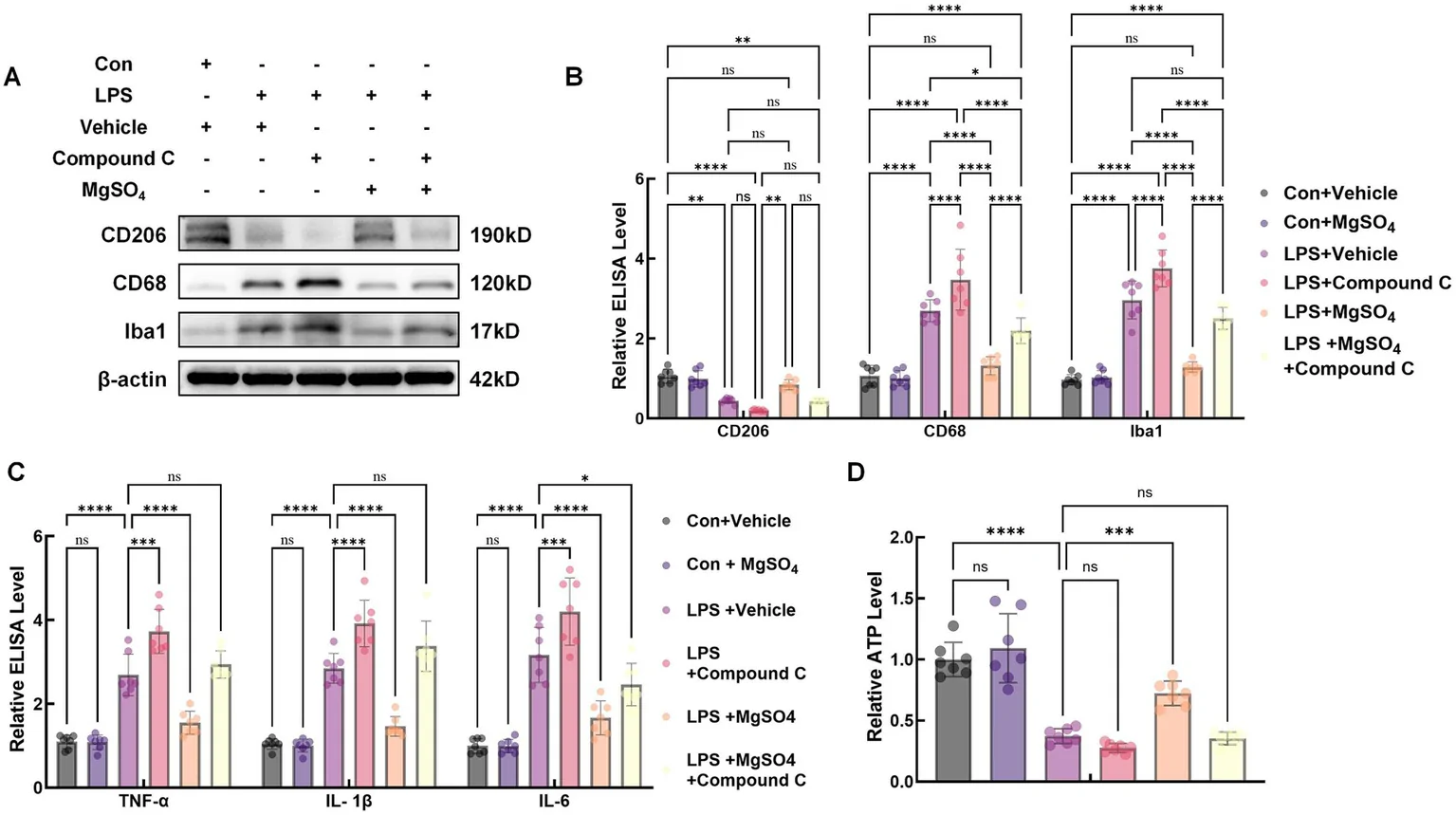
Compound C partially attenuates the anti-inflammatory and metabolic effects associated with MgSO₄ treatment in LPS-treated BV2 microglia. **(A,B)** Representative western blots and ELISA analysis of CD206, CD68, and Iba1 (*n* = 7 biological samples per group). **(C)** ELISA measurement of TNF-α, IL-1β, and IL-6 (*n* = 6 per group). **(D)** Relative intracellular ATP readouts (*n* = 6 per group). Data are presented as mean ± SD. Statistical significance was determined using one-way ANOVA followed by Tukey’s multiple comparisons test for comparisons among multiple groups. Behavioral data obtained from repeated measurements were analyzed using two-way repeated-measures ANOVA followed by Šídák’s multiple comparisons test. *p < 0.05, **p < 0.01, ***p < 0.001, ****p < 0.0001; ns, not significant.

Total AMPK expression remained relatively stable across treatment groups, whereas LPS reduced p-AMPK, PINK1, and Parkin. MgSO₄ restored these proteins, while Compound C partially attenuated the MgSO₄-associated recovery (Figures 7A,B). LPS also decreased TOMM20 and increased DRP1. MgSO₄ partially normalized both mitochondrial markers, whereas Compound C diminished these effects (Figures 7C,D). These findings support pharmacological involvement of AMPK signaling in the mitochondrial and anti-inflammatory effects associated with MgSO₄ treatment.

**Figure 7:**
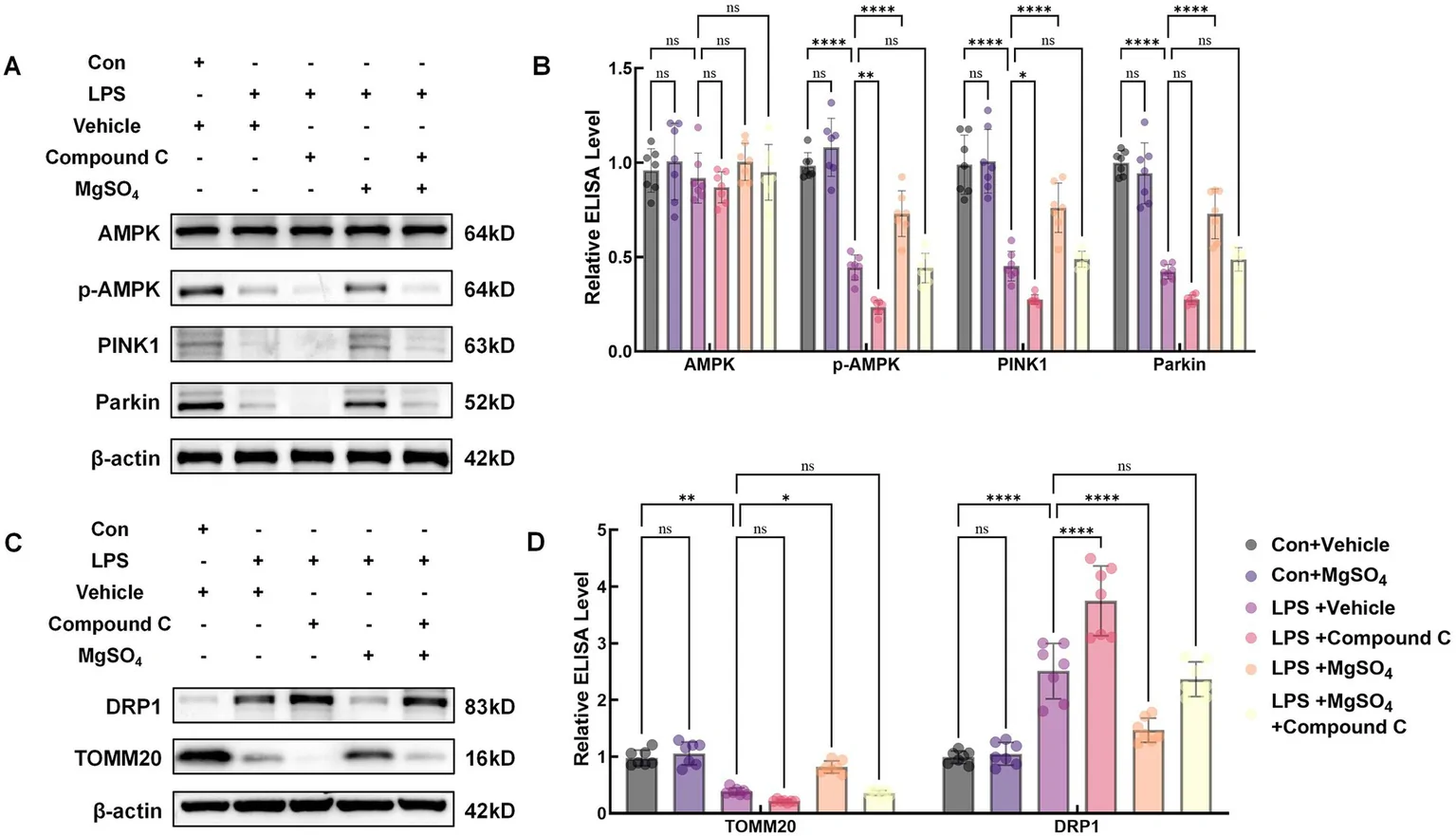
Compound C partially attenuates MgSO_4_-associated restoration of AMPK/PINK1/Parkin signaling and mitochondrial markers in LPS-treated BV2 microglia. **(A,B)** Representative western blots and ELISA analysis of total AMPK, p-AMPK, PINK1, and Parkin (*n* = 7 biological samples per group). **(C,D)** Representative western blots and densitometric analysis of DRP1 and TOMM20 (*n* = 3 biological samples per group). Compound C was used as a pharmacological AMPK inhibitor, and β-actin served as the internal control. Data are presented as mean ± SD. Statistical significance was determined using one-way ANOVA followed by Tukey’s multiple comparisons test for comparisons among multiple groups. Behavioral data obtained from repeated measurements were analyzed using two-way repeated-measures ANOVA followed by Šídák’s multiple comparisons test. *p < 0.05, **p < 0.01, ***p < 0.001, ****p < 0.0001; ns, not significant.

### 3.5 Compound C partially attenuates the neuroprotective effects of MgSO₄ in postoperative mice

Surgery increased hippocampal TNF-α, IL-1β, and IL-6, whereas MgSO₄ markedly reduced these inflammatory responses. Compound C partially attenuated the anti-inflammatory effects associated with MgSO₄ treatment (Figure 8A). Surgery also reduced CD206 while increasing CD68 and Iba1. MgSO₄ partially normalized these microglial activation-associated markers, whereas Compound C diminished this effect (Figures 8B,C). Total AMPK expression showed little change among groups, whereas surgery reduced p-AMPK, PINK1, and Parkin. MgSO₄ restored these signaling proteins, while Compound C partially attenuated their recovery (Figures 8D,E).

**Figure 8:**
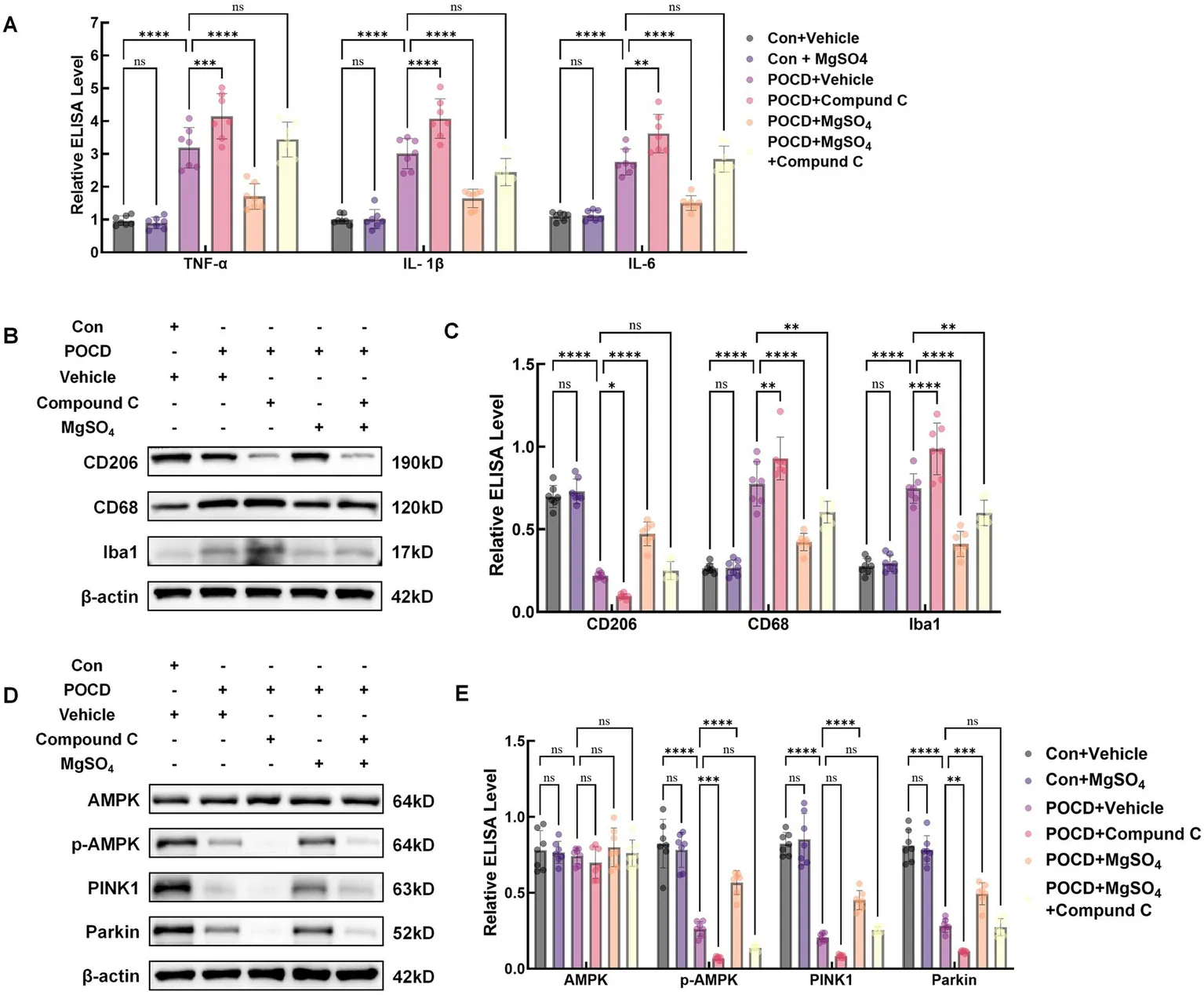
Pharmacological AMPK inhibition attenuates the anti-inflammatory and signaling effects associated with MgSO_4_ after surgery. **(A)** ELISA measurement of TNF-α, IL-1*β*, and IL-6 in hippocampal tissue (*n* = 6 mice per group). **(B,C)** Representative western blots and ELISA analysis of CD206, CD68, and Iba1 (*n* = 7 biological samples per group). **(D,E)** Representative western blots and ELISA analysis of total AMPK, p-AMPK, PINK1, and Parkin (*n* = 7 biological samples per group). Compound C was used as a pharmacological AMPK inhibitor, and β-actin served as the internal control. Data are presented as mean ± *SD*. Statistical significance was determined using one-way ANOVA followed by Tukey’s multiple comparisons test for comparisons among multiple groups. Behavioral data obtained from repeated measurements were analyzed using two-way repeated-measures ANOVA followed by Šídák’s multiple comparisons test. *p < 0.05, **p < 0.01, ***p < 0.001, ****p < 0.0001; ns, not significant.

Behaviorally, surgery increased escape latency and the number of errors in the Morris water maze. MgSO₄ improved both outcomes, whereas Compound C reduced the magnitude of this improvement (Figures 9A,B). Surgery also reduced novel-object exploration time and the discrimination rate, both of which were improved by MgSO₄ and partially attenuated by Compound C (Figure 9C). In the open-field test, MgSO₄ partially restored surgery-associated reductions in total distance traveled and central-zone entries, whereas Compound C diminished this behavioral improvement (Figure 9D).

**Figure 9:**
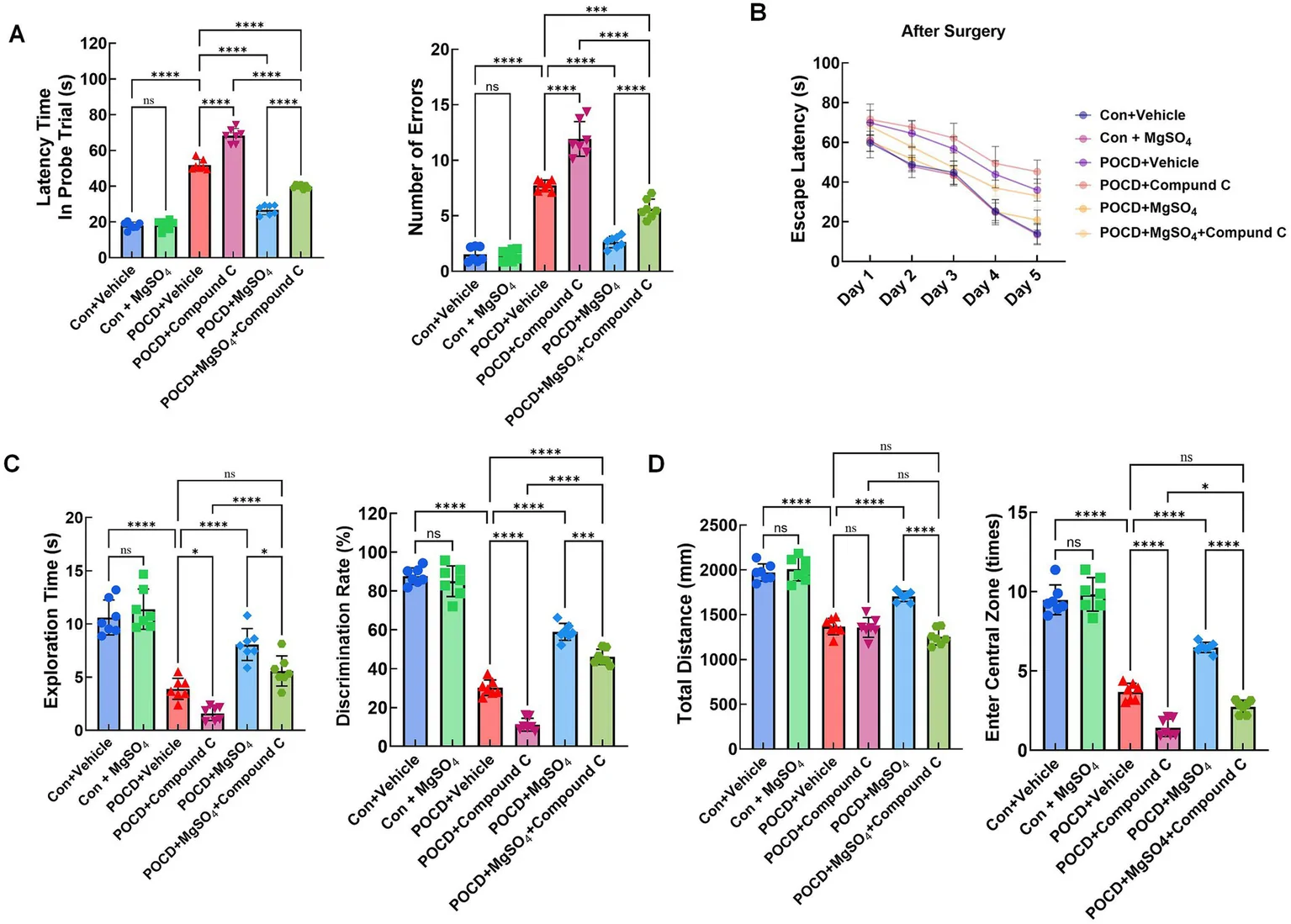
Compound C partially attenuates the behavioral protection associated with MgSO_4_ in postoperative mice. **(A)** MWM probe-trial latency and error measures on POD6. **(B)** Escape-latency learning curves during MWM acquisition from POD1 to POD5. **(C)** Exploration time and discrimination rate in the NOR test on POD9. **(D)** Total distance traveled and central-zone entries in the OFT on POD7. Behavioral analyses include *n* = 6 mice per group. Data are presented as mean ± SD. Statistical significance was determined using one-way ANOVA followed by Tukey’s multiple comparisons test for comparisons among multiple groups. Behavioral data obtained from repeated measurements were analyzed using two-way repeated-measures ANOVA followed by Šídák’s multiple comparisons test. *p < 0.05, **p < 0.01, ***p < 0.001, ****p < 0.0001; ns, not significant.

## 4 Discussion

This study shows that MgSO₄ ameliorates postoperative cognitive deficits, hippocampal neuroinflammation, and mitochondrial quality-control abnormalities in experimental POCD.

Because Morris water-maze training was initiated during the early postoperative period, residual postoperative pain and surgery-related motor impairment may have influenced behavioral performance. Although perioperative analgesia was provided and locomotor activity was assessed using the open-field test, these factors cannot be completely excluded. To further address this concern, swimming speed during MWM acquisition was analyzed, and no significant differences were observed among groups, suggesting that altered swimming ability was unlikely to be the primary contributor to the differences in escape latency. Nevertheless, because behavioral assessments were performed during the early postoperative period after tibial fracture surgery, subtle effects of postoperative discomfort or transient functional limitations may still have influenced some behavioral readouts. Therefore, the behavioral findings should be interpreted as postoperative neurobehavioral impairment associated with cognitive dysfunction rather than cognitive dysfunction in isolation.

Surgery was associated with increased inflammatory cytokines, an altered microglial activation-associated marker profile, alterations in mitochondrial dynamics-related proteins, reduced ATP levels, reduced p-AMPK, reduced PINK1/Parkin and LC3-II, and p62 accumulation. MgSO₄ partially reversed these changes and improved behavioral outcomes. Similar effects were reproduced in LPS-treated BV2 microglia, in which MgSO₄ improved ATP levels and mitochondrial membrane potential and restored a PINK1/Parkin-associated mitophagy-related protein profile. Importantly, Compound C partially attenuated the molecular, inflammatory, metabolic, and behavioral effects of MgSO₄, supporting involvement of AMPK signaling in this protective response.

Open tibial fracture combined with isoflurane anesthesia is widely used to model perioperative cognitive impairment and associated neuroinflammation (Lu et al., 2024). Related surgery/anesthesia models can produce deficits in spatial learning, episodic memory, and recognition memory (Liu et al., 2021; Gao et al., 2021). Consistent with these observations, postoperative mice in the present study exhibited abnormalities in the Morris water maze, novel object recognition test, and open field test. Surgery and anesthesia are also known to induce central inflammatory responses and mitochondrial dysfunction (Gao et al., 2021; Zhang Z. et al., 2024; Ding et al., 2022). Our *in vivo* data showed hippocampal inflammatory and mitochondrial abnormalities, while the LPS-stimulated BV2 model provided a complementary cellular system for examining the microglial response (Ji et al., 2024). In this cellular model, LPS reduced PINK1, Parkin, and LC3-II and increased p62, whereas MgSO₄ reversed these changes while improving ATP levels and mitochondrial membrane potential. Thus, the present data support restoration, rather than suppression, of the PINK1/Parkin-associated mitophagy-related protein profile by MgSO₄ under inflammatory stress.

Perioperative magnesium homeostasis has been linked to cognitive outcomes, and hypomagnesemia is common in hospitalized and older populations (Boccardi et al., 2023). Observational studies have associated lower magnesium status with delirium or cognitive impairment, while nutritional and prospective cohort studies also support relationships between magnesium status and cognitive health (Boccardi et al., 2023; Lo et al., 2019; Chen et al., 2021). At the mechanistic level, magnesium can modulate N-methyl-D-aspartate receptor activity, calcium influx, oxidative stress, and inflammatory signaling. MgSO₄ has been shown to suppress microglial inflammatory responses and preserve cellular bioenergetic function in experimental systems (Gao et al., 2013; Wang et al., 2025; Clerc et al., 2013; Le Dieu-Lugon et al., 2021; Patel et al., 2024). These properties provide a plausible basis for the reductions in hippocampal cytokines and the restoration of ATP levels observed in the present study.

Mitochondrial dysfunction is increasingly recognized as an important link between neuroinflammation and cognitive impairment in POCD (Zhang Z. et al., 2024; Liu et al., 2025). Mitophagy is a major component of mitochondrial quality control, and PINK1/Parkin-dependent mitophagy can limit the accumulation of damaged mitochondria and restrain inflammatory signaling (Zhang et al., 2025a; Yang et al., 2024; Wang et al., 2023). The direction of the present results is consistent across the *in vivo* and *in vitro* models: POCD or LPS reduced PINK1, Parkin, and LC3-II and increased p62, while MgSO₄ reversed this pattern. Because the present study assessed mitophagy-related protein expression rather than autophagic flux directly, these findings are interpreted as restoration of a PINK1/Parkin-associated mitochondrial quality-control profile rather than definitive demonstration of increased mitophagic flux.

AMPK is a central metabolic sensor that can interact with mitochondrial quality-control pathways, including PINK1/Parkin-dependent mitophagy (Seabright and Lai, 2020). In the present study, MgSO₄ restored AMPK phosphorylation in hippocampal tissue and BV2 cells, whereas Compound C partially diminished the effects of MgSO₄ on PINK1/Parkin signaling, mitochondrial markers, ATP levels, inflammatory responses, and behavior. These findings support a model in which AMPK activation contributes to the neuroprotective effects of MgSO₄ and is functionally linked to restoration of PINK1/Parkin-associated mitochondrial quality control. However, Compound C is a pharmacological inhibitor and is not fully selective for AMPK; therefore, the pathway relationship should be interpreted as pharmacological evidence rather than definitive genetic proof of causality.

This study has several limitations. First, AMPK involvement was evaluated pharmacologically with Compound C, and genetic approaches such as AMPK knockdown or conditional deletion were not performed. Furthermore, BV2 cells are an immortalized microglial model and may not fully reproduce the phenotype of primary microglia. In addition, only male mice were studied, and the use of young adult animals limits assessment of age- and sex-dependent responses that may be relevant to clinical POCD. ATP measurements and JC-1 staining provide information regarding cellular energy status and mitochondrial membrane potential, respectively, but do not directly assess mitochondrial respiratory capacity or oxidative phosphorylation efficiency. Therefore, although these findings suggest improved mitochondrial-related metabolic status after MgSO₄ treatment, direct evaluation of mitochondrial respiration using approaches such as Seahorse extracellular flux analysis will be necessary in future studies. The present study assessed mitophagy-related protein expression rather than directly measuring autophagic flux. Therefore, the observed changes should be interpreted as restoration of a PINK1/Parkin-associated mitochondrial quality-control profile rather than definitive evidence of increased mitophagic flux. Although perioperative buprenorphine analgesia, sham controls, and swimming speed/locomotor assessments were used, residual pain, inflammation, or motor impairment cannot be completely excluded as contributors to early postoperative behavioral performance, and direct pain-threshold and cytokine time-course data are lacking. Finally, the study focused primarily on early postoperative changes and did not systematically assess long-term cognitive outcomes or neuroplasticity. These limitations should be addressed in future studies using primary or genetically defined microglial models, cell-type-specific approaches, and longer-term behavioral assessment.

## 5 Conclusion

MgSO₄ alleviated neuroinflammation and cognitive dysfunction in experimental POCD while improving hippocampal mitochondrial quality-control abnormalities and microglial activation-associated marker changes. In postoperative mice and LPS-treated BV2 microglia, disease-related stress reduced p-AMPK, PINK1, Parkin, and LC3-II and increased p62; MgSO₄ partially reversed these alterations, restored ATP-related readouts,and improved mitochondrial stress-associated alterations. The partial loss of these effects after Compound C treatment supports involvement of AMPK signaling in the MgSO₄ response. Collectively, these findings support a model in which AMPK/PINK1/Parkin-related mitochondrial quality control is involved in the neuroprotective effects of MgSO₄ in experimental POCD and warrants further mechanistic and translational investigation.

## Data Availability

The raw data supporting the conclusions of this article will be made available by the authors, without undue reservation.
