# Supplementary material for: AMPK/PINK1/Parkin pathway-associated mitochondrial quality control contributes to magnesium sulfate-induced neuroprotection in postoperative cognitive dysfunction
Source: Front Cell Neurosci. 2026 Oct 28;20:1969109. doi: 10.3389/fncel.2026.1969109 (PMC13751457; doi:10.3389/fncel.2026.1969109)

Supplementary Material

AMPK/PINK1/Parkin Pathway-Associated Mitochondrial Quality Control Contributes to Magnesium Sulfate-Induced Neuroprotection in Postoperative Cognitive Dysfunction

This supplementary file contains the antibody information used for western blot analysis and the original full-length western blot images corresponding to the cropped immunoblots presented in the main manuscript. For the original full-length blots, only non-informative black borders were trimmed; marker lanes and all experimental lanes were retained.

Supplementary Table 1. Antibodies used for western blot analysis

Antibody	Manufacturer	Catalog number	Dilution
PINK1	Proteintech	23274-1-AP	1:1,000
Parkin/PARK2	Proteintech	14060-1-AP	1:1,000
AMPKα	Cell Signaling Technology	2532	1:1,000
phospho-AMPKα (Thr172)	Cell Signaling Technology	2535	1:1,000
CD68	Proteintech	28058-1-AP	1:1,000
Iba1/AIF1	Proteintech	10904-1-AP	1:1,000
CD206/MRC1	Proteintech	18704-1-AP	1:1,000
Bax	Proteintech	50599-2-Ig	1:2,000
Bcl-2	Proteintech	68103-1-Ig	1:2,000
DRP1/DNM1L	Proteintech	12957-1-AP	1:1,000
OPA1	Proteintech	27733-1-AP	1:1,000
TOMM20	Proteintech	11802-1-AP	1:1,000
LC3B	Proteintech	18725-1-AP	1:1,000
P62/SQSTM1	Proteintech	18420-1-AP	1:1,000
β-actin	Proteintech	66009-1-Ig	1:5,000
HRP-conjugated Goat Anti-Mouse IgG (H+L)	Proteintech	SA00001-1	1:5,000
HRP-conjugated Goat Anti-Rabbit IgG (H+L)	Proteintech	SA00001-2	1:5,000

Primary and secondary antibodies were used at the indicated dilutions for western blot analysis.

Supplementary Figure 1. Full-length western blot images corresponding to Figure 1.

Original full-length western blot images corresponding to the representative immunoblots presented in Figure 1. Molecular weight markers and all experimental lanes were retained.

figure1 B

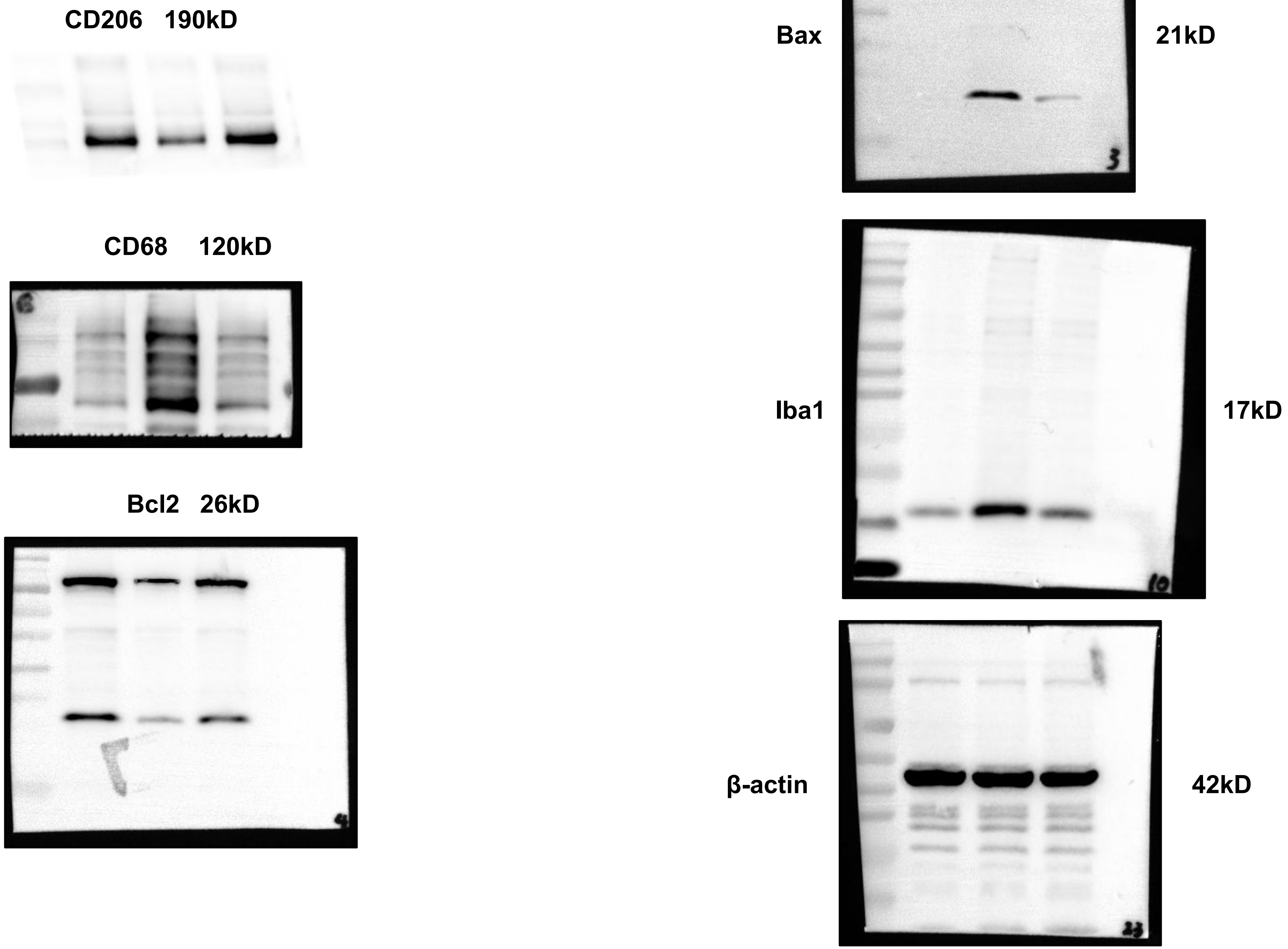
CD206 190kD
CD68 120kD
Bcl2 26kD
Bax
21kD
Iba1
17kD
β-actin
42kD

figure1 D

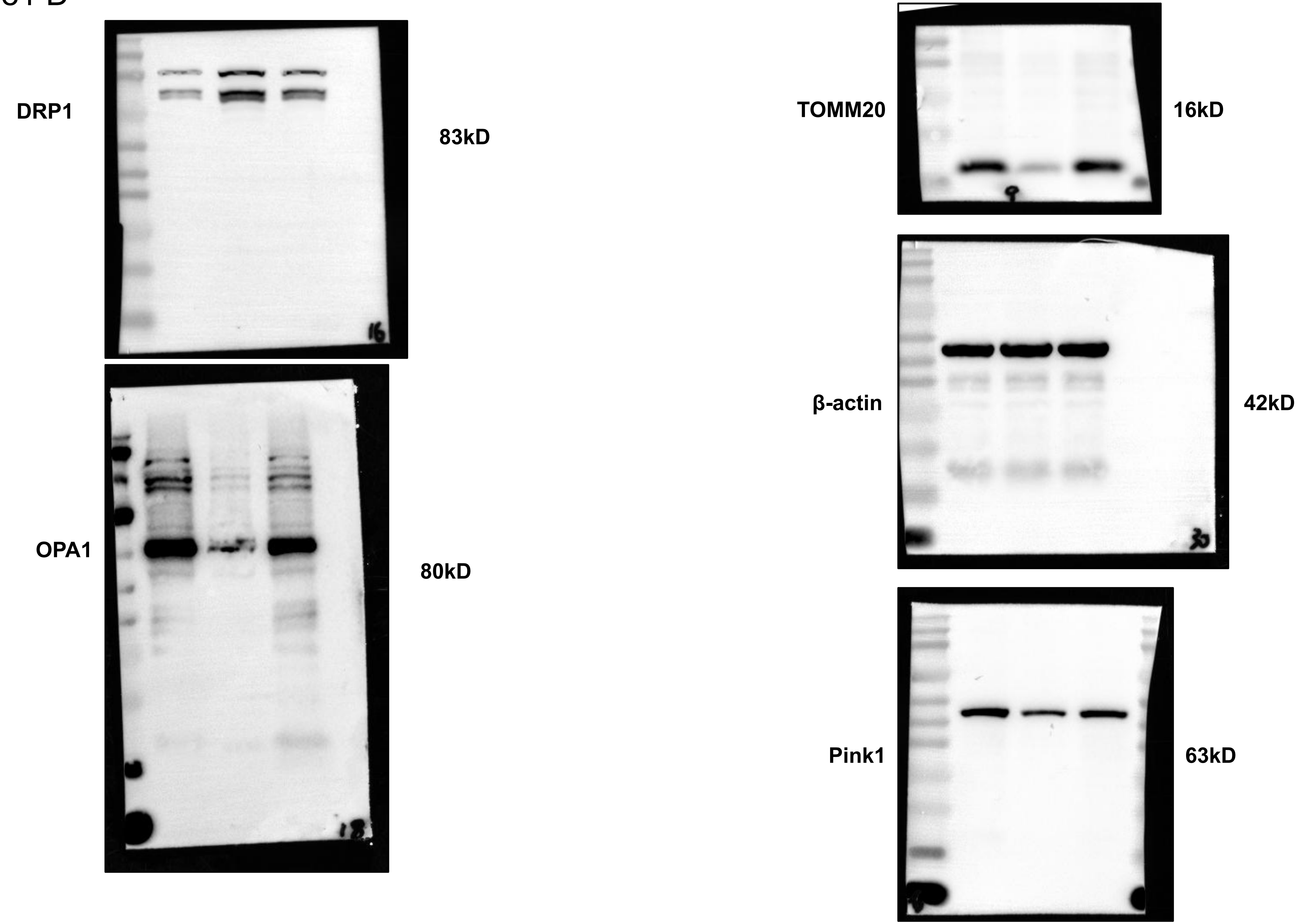

figure1 D

P62 62kD

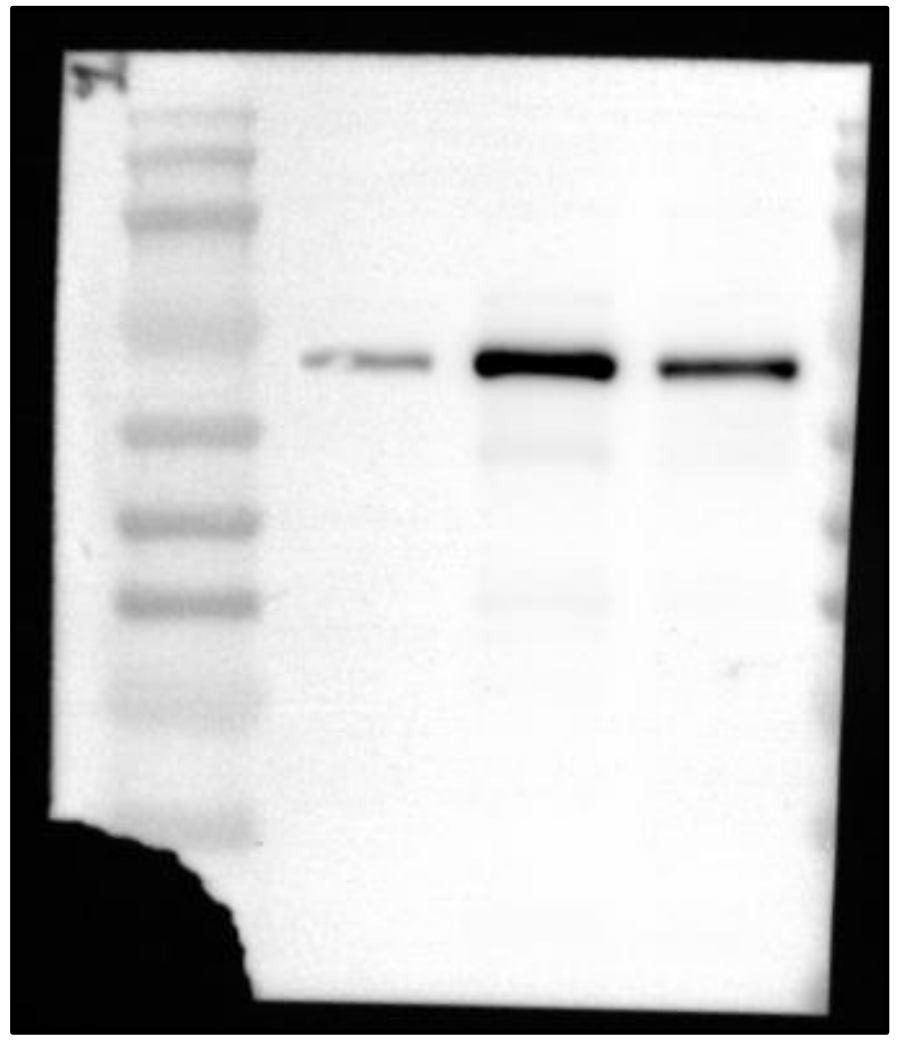

LC3-II 16kD

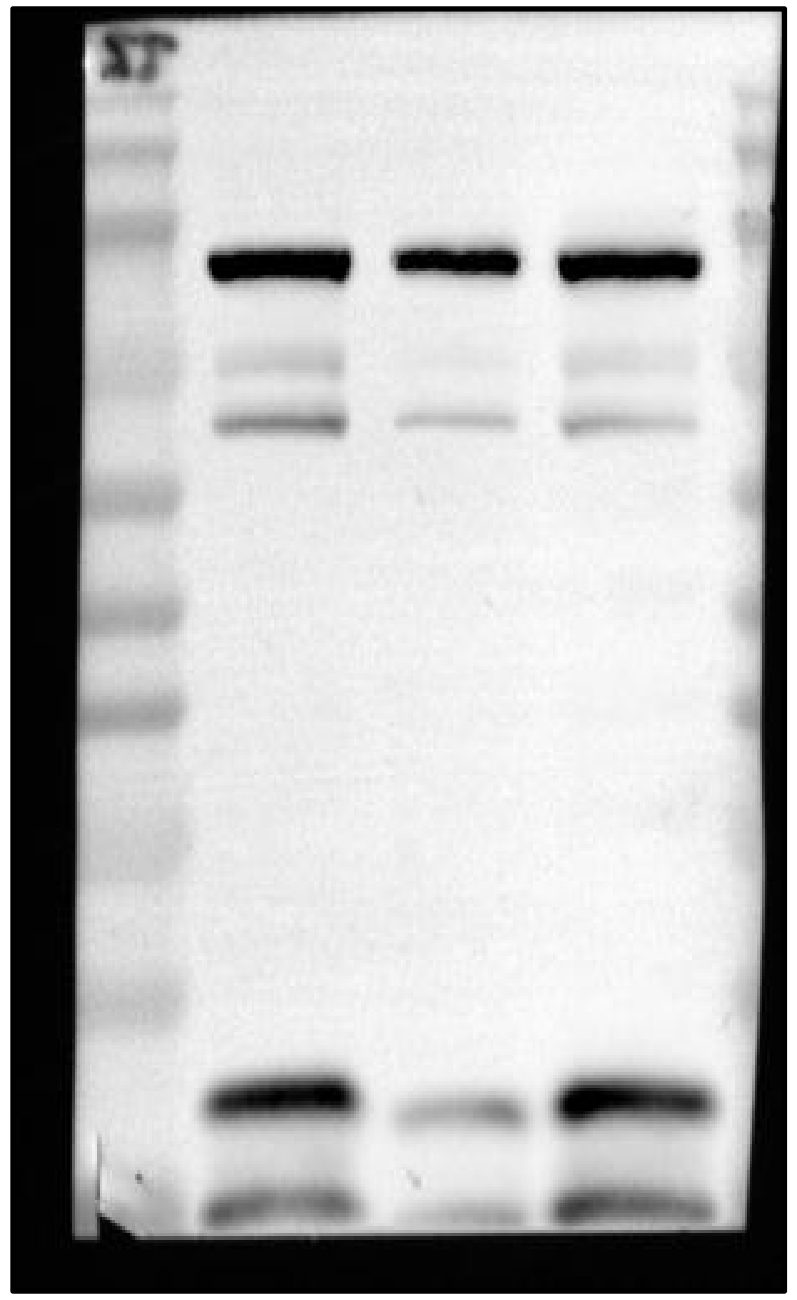

Parkin 52kD

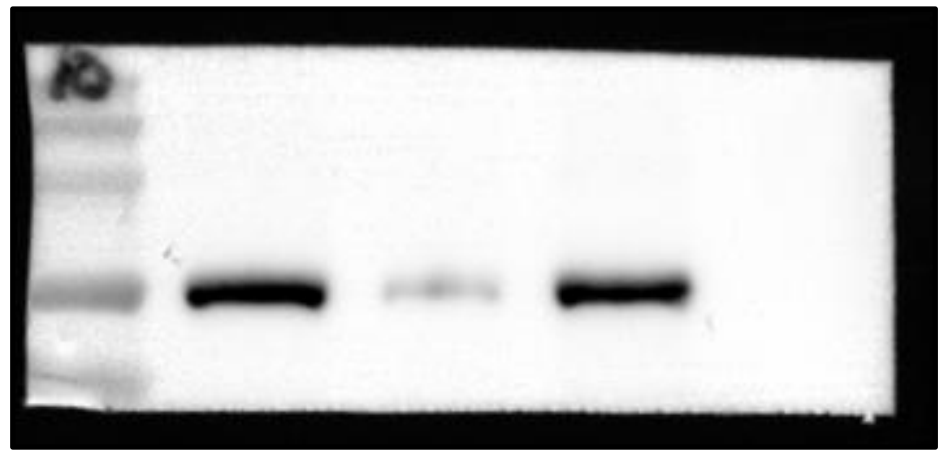

β-actin 42kD

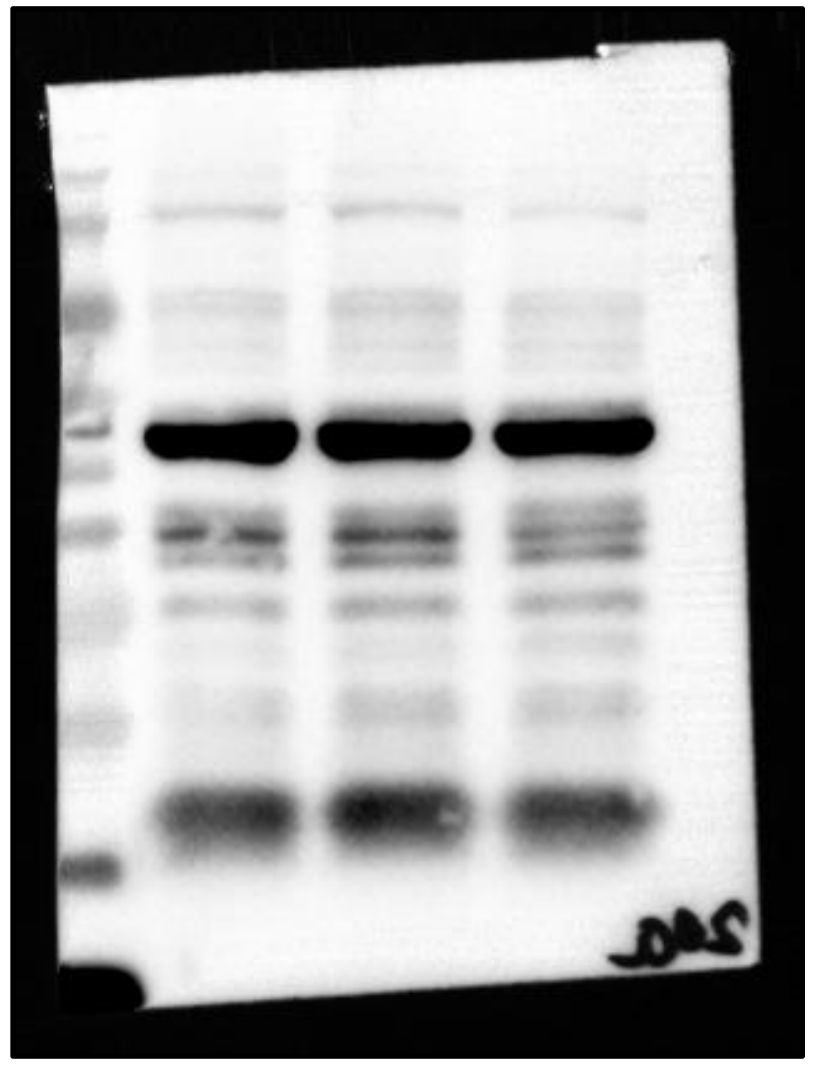

Supplementary Figure 2. Full-length western blot images corresponding to Figure 2.

Original full-length western blot images corresponding to the representative immunoblots presented in Figure 2. Molecular weight markers and all experimental lanes were retained.

figure2 A

AMPK 64kD

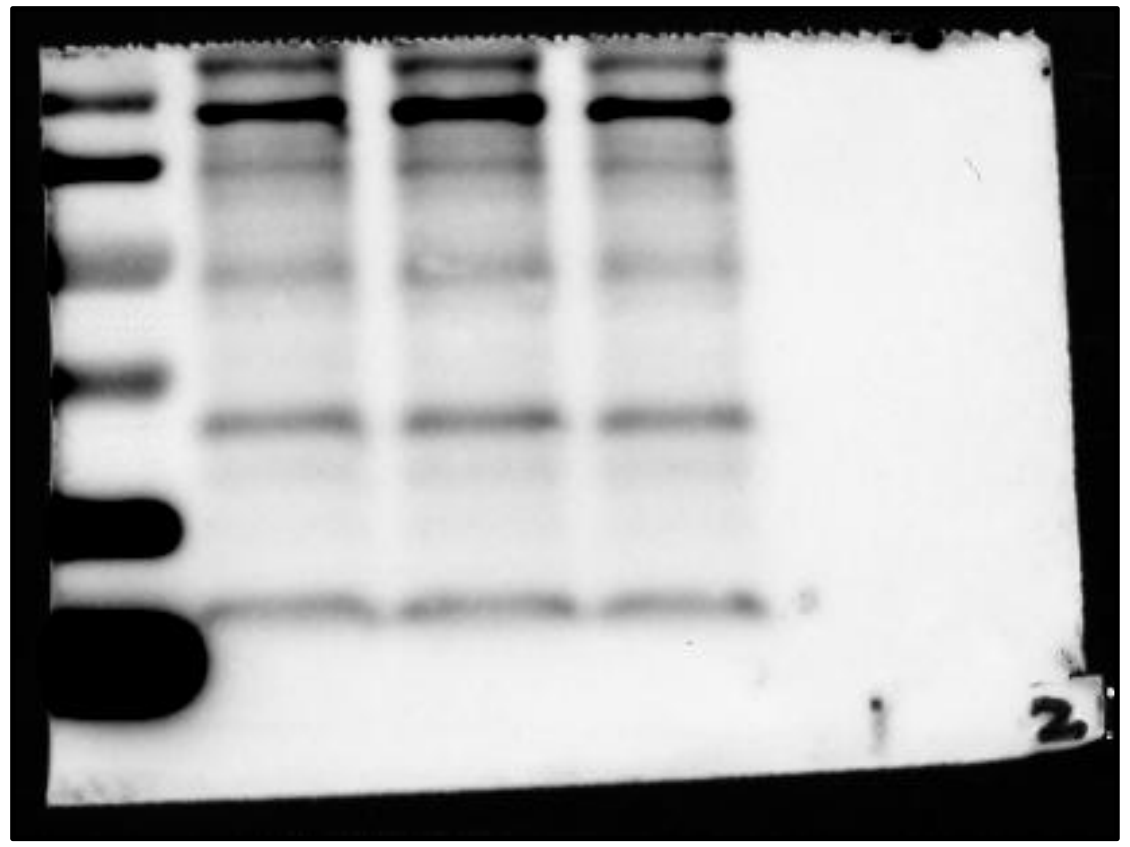

p-AMPK 62kD

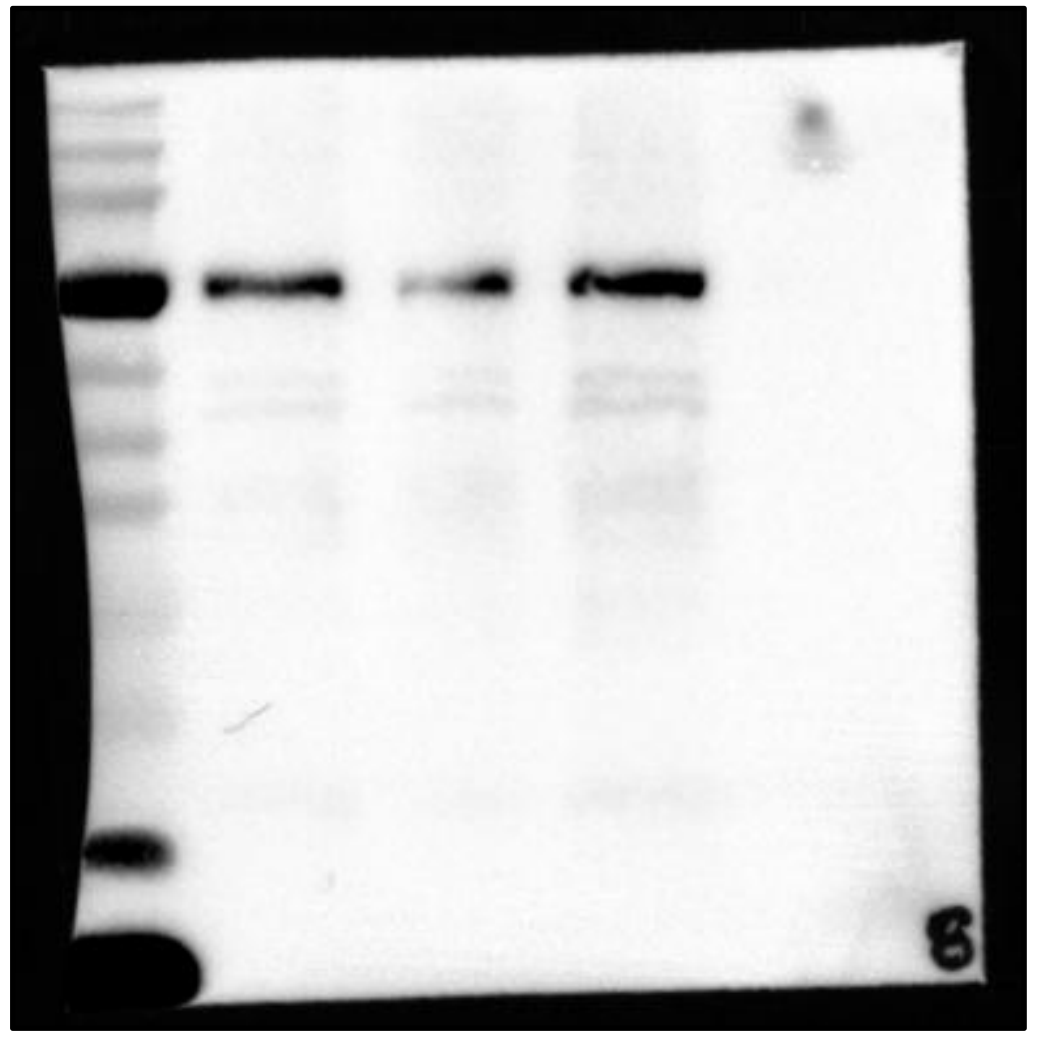

β-actin 42kD

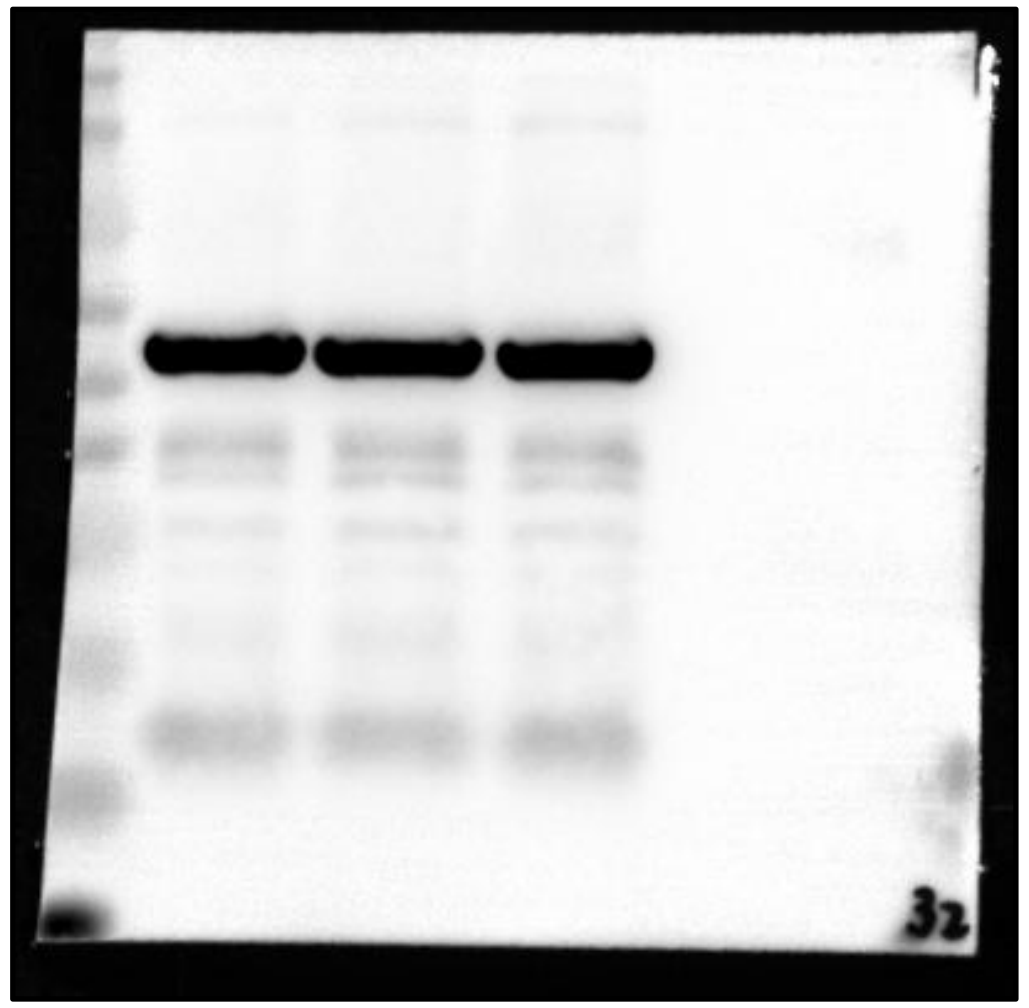

Supplementary Figure 3. Full-length western blot images corresponding to Figure 3.

Original full-length western blot images corresponding to the representative immunoblots presented in Figure 3. Molecular weight markers and all experimental lanes were retained.

figure3 A

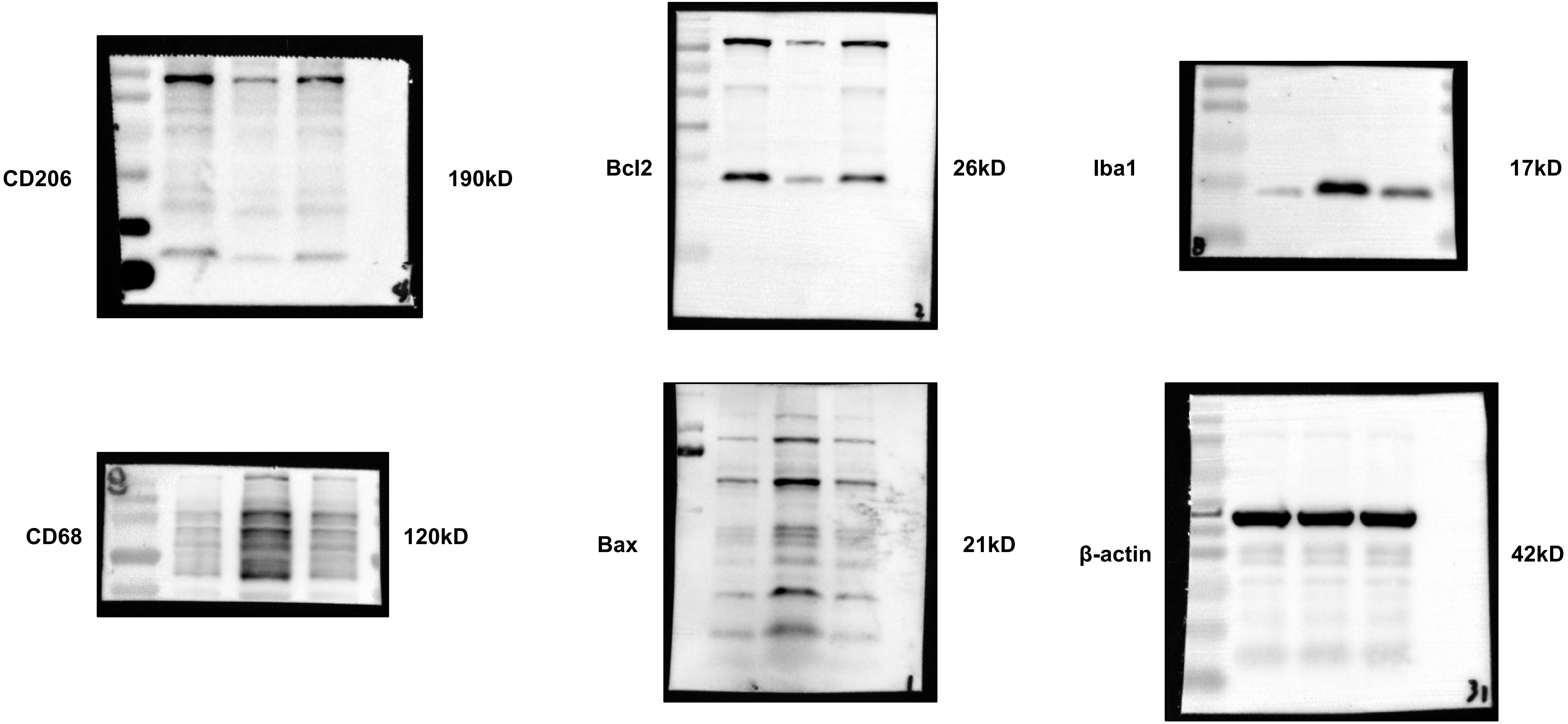
CD206
190kD
Bcl2
26kD
Iba1
17kD
CD68
120kD
Bax
21kD
β-actin
42kD

figure3 C

AMPK

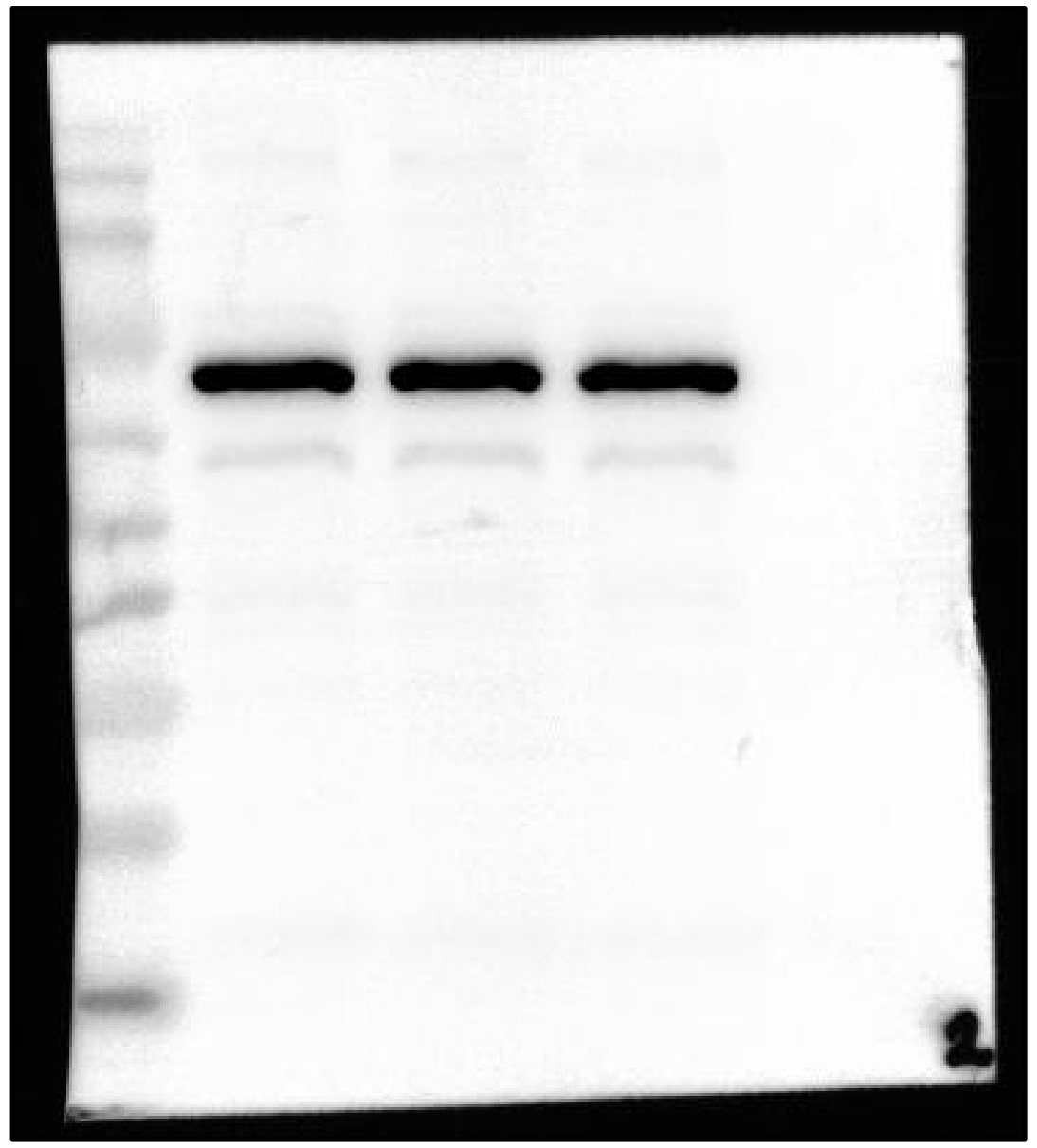

64kD

β-actin

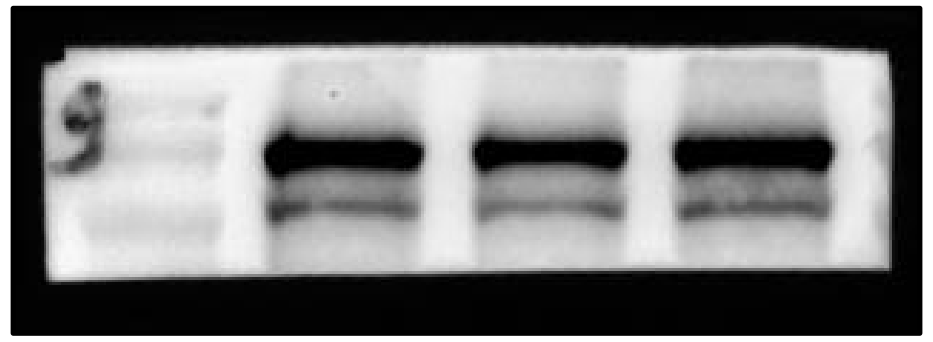

42kD

p-AMPK

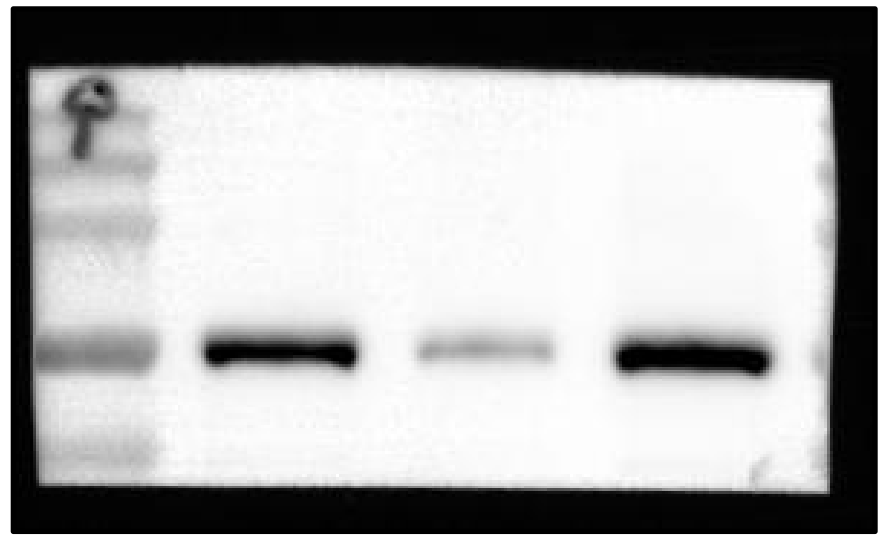

62kD

Supplementary Figure 4. Full-length western blot images corresponding to Figure 4.

Original full-length western blot images corresponding to the representative immunoblots presented in Figure 4. Molecular weight markers and all experimental lanes were retained.

figure4 D

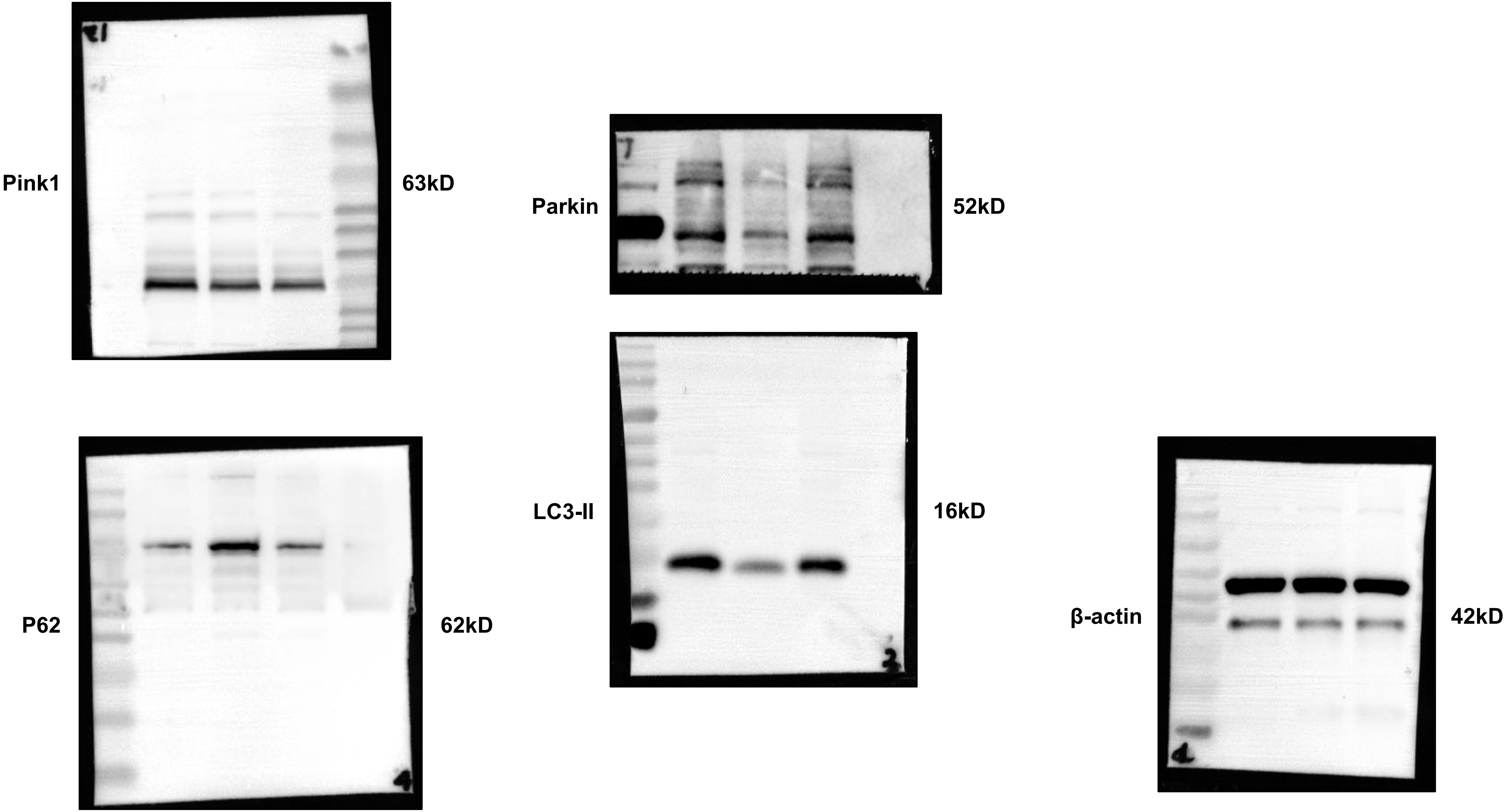

Supplementary Figure 5. Full-length western blot images corresponding to Figure 5.

Original full-length western blot images corresponding to the representative immunoblots presented in Figure 5. Molecular weight markers and all experimental lanes were retained.

figure5A

CD206 190kD

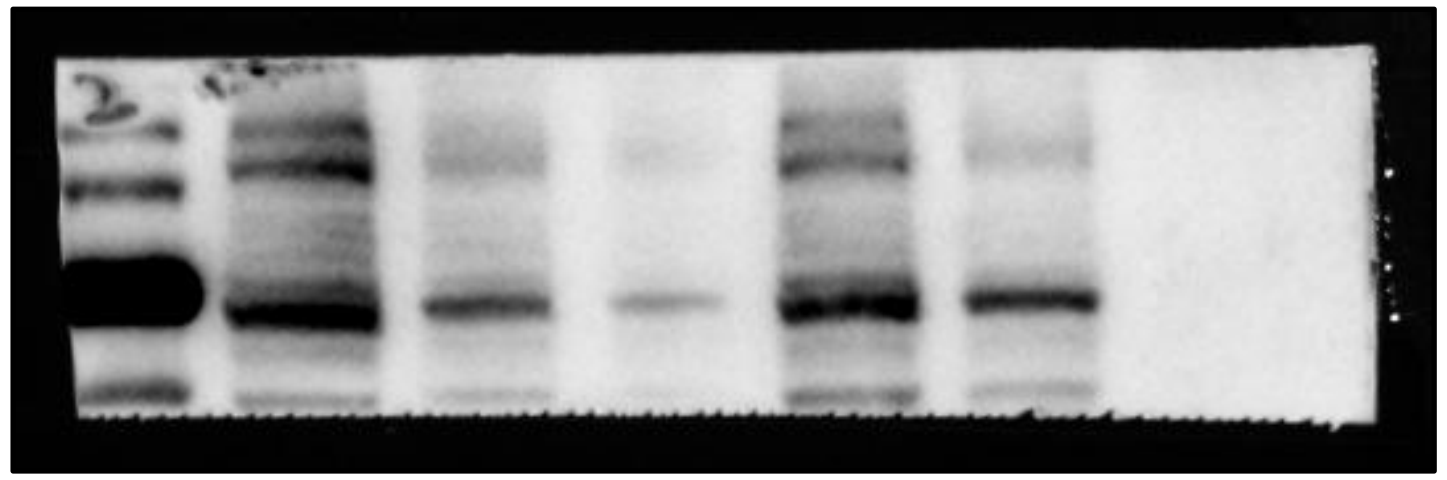

CD68 120kD

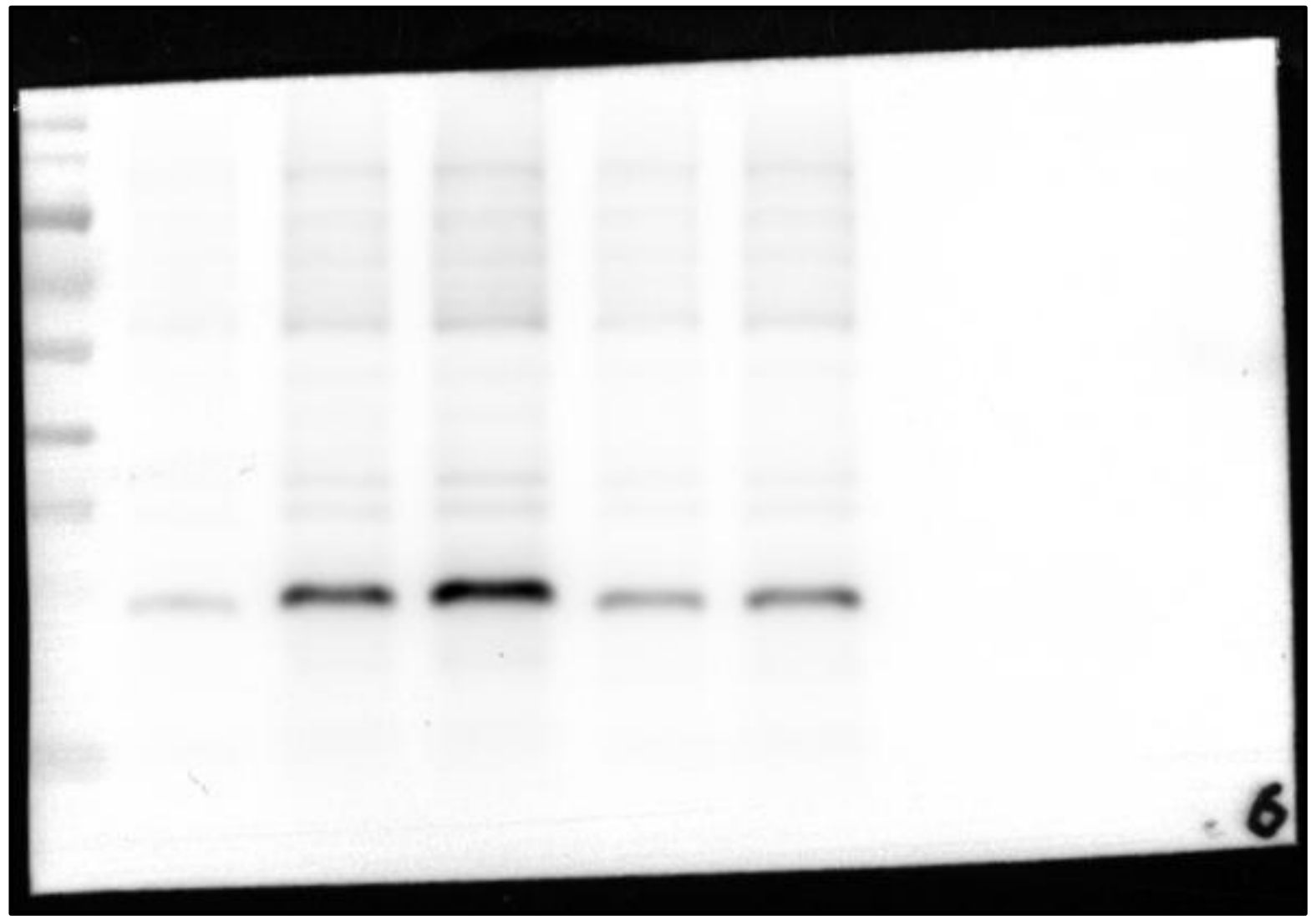

Iba1 17kD

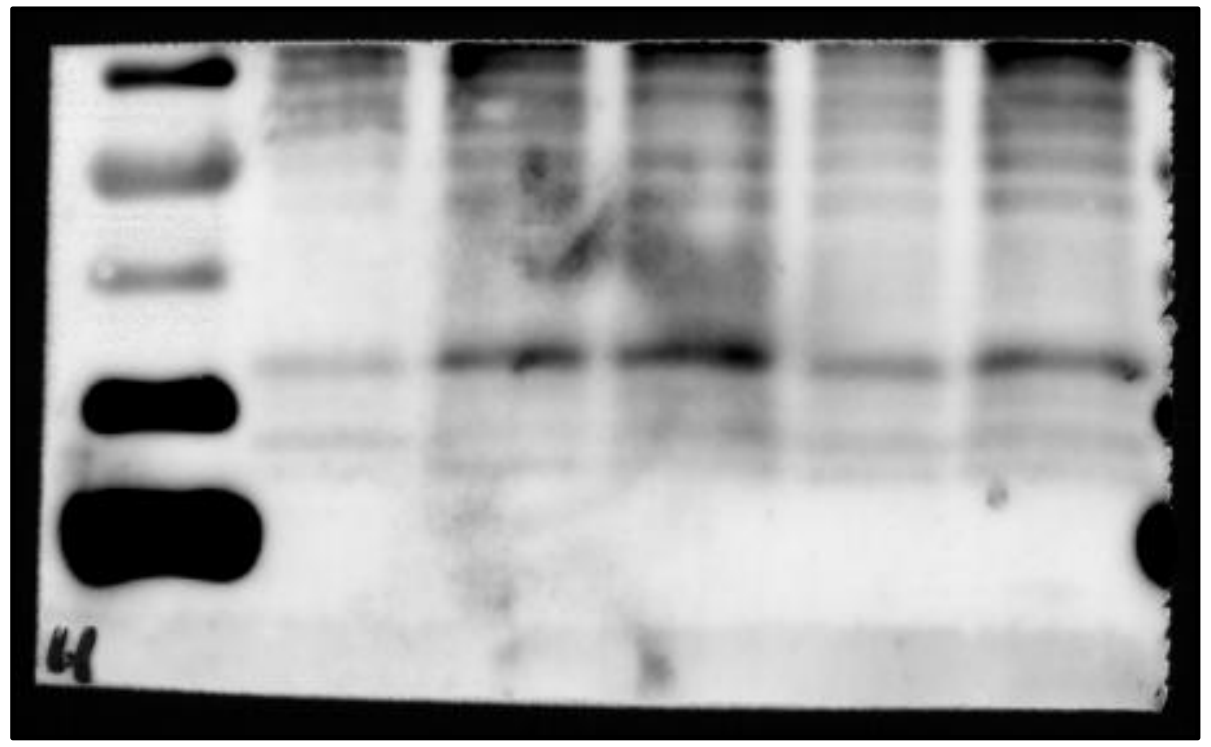

β-actin 42kD

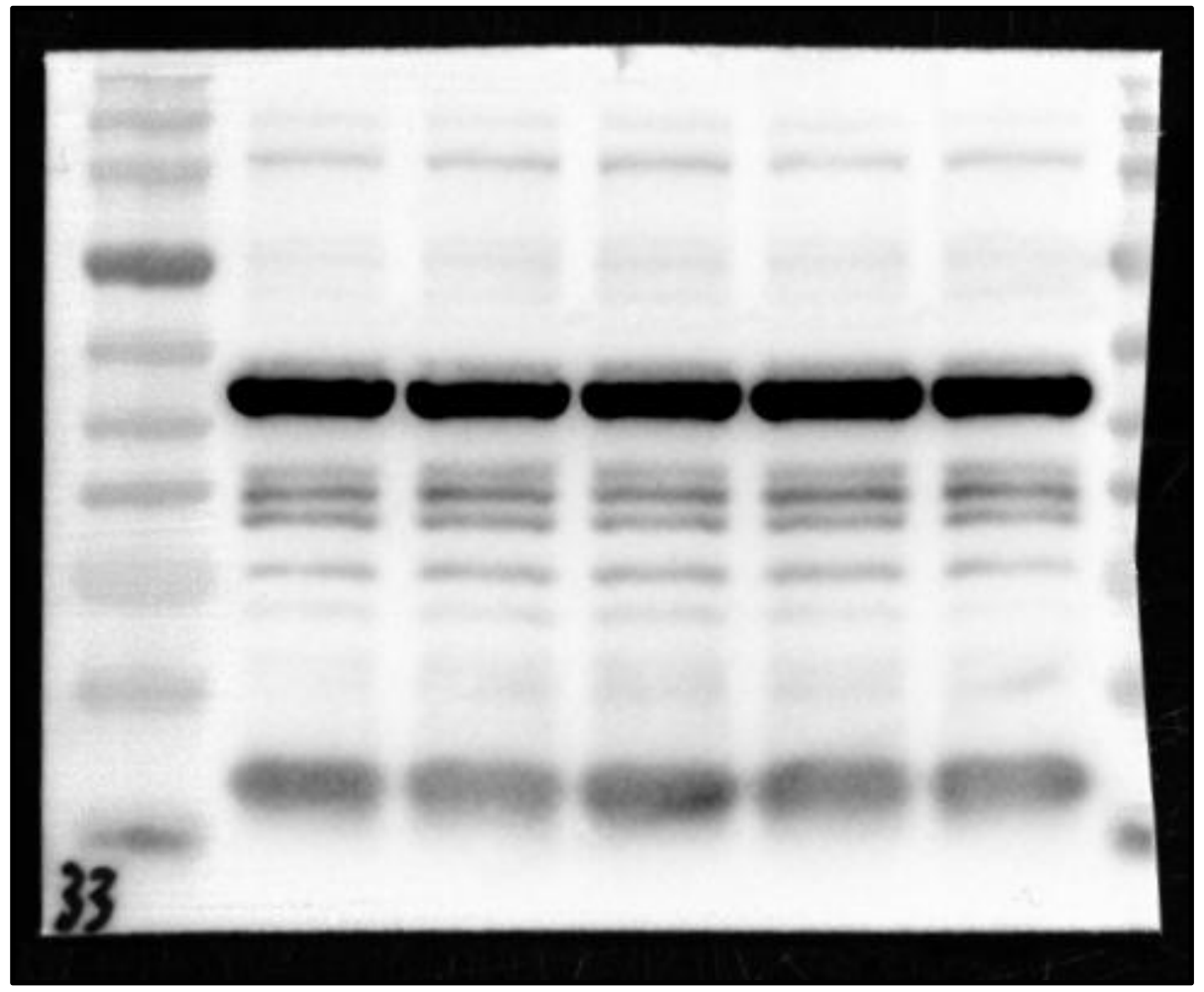

Supplementary Figure 6. Full-length western blot images corresponding to Figure 6.

Original full-length western blot images corresponding to the representative immunoblots presented in Figure 6. Molecular weight markers and all experimental lanes were retained.

figure6 A

AMPK

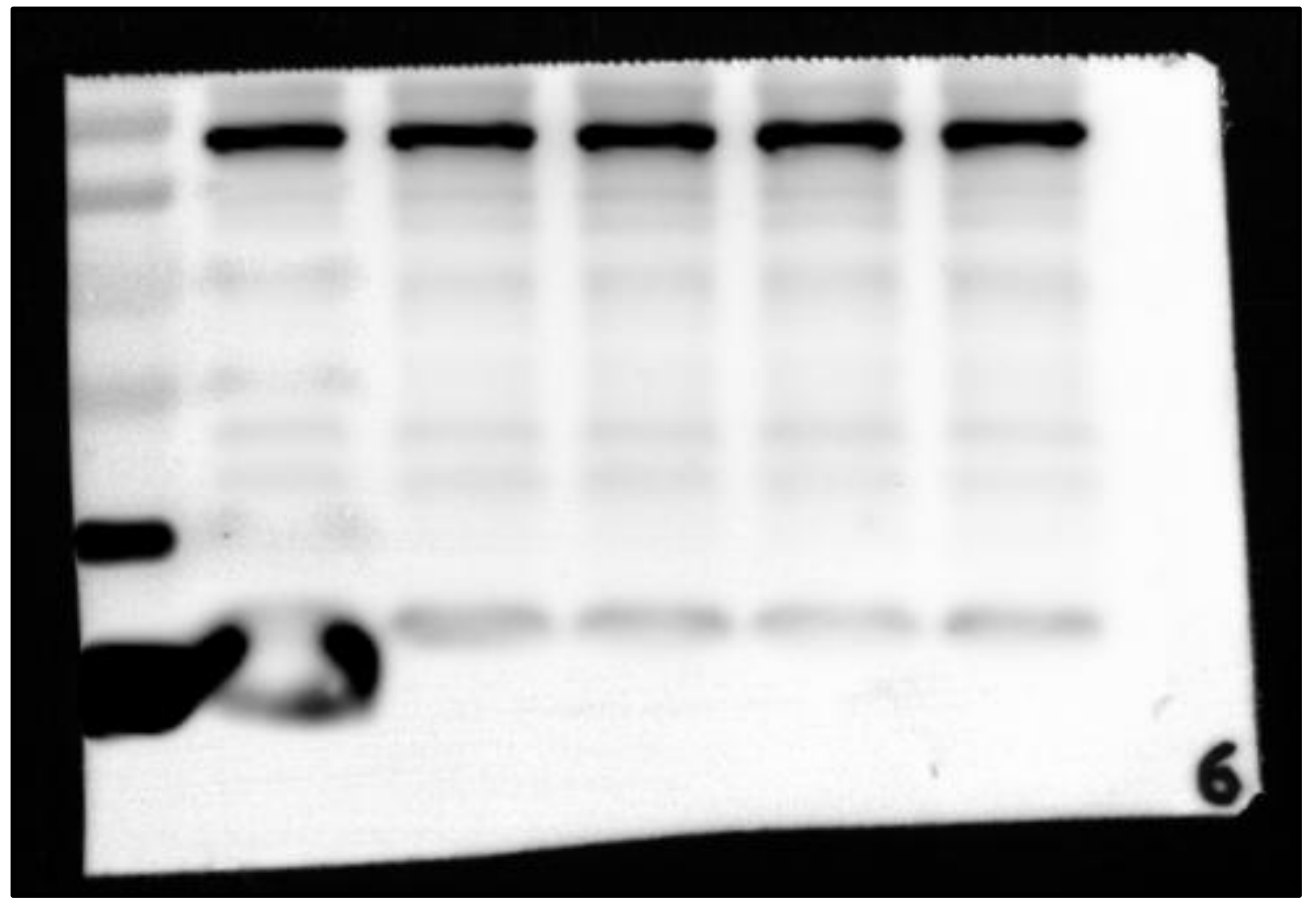

64kD

p-AMPK

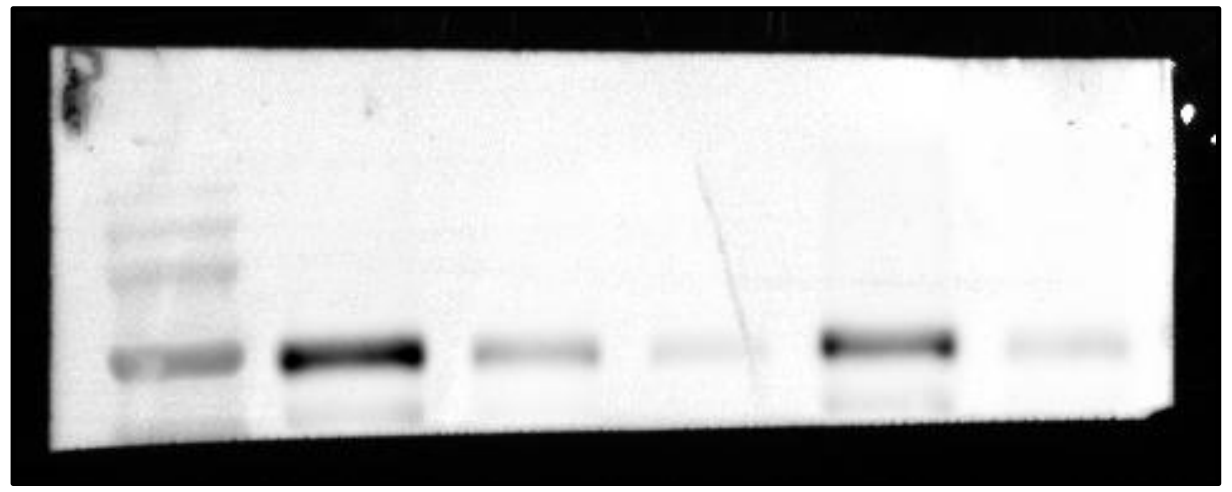

62kD

Pink1

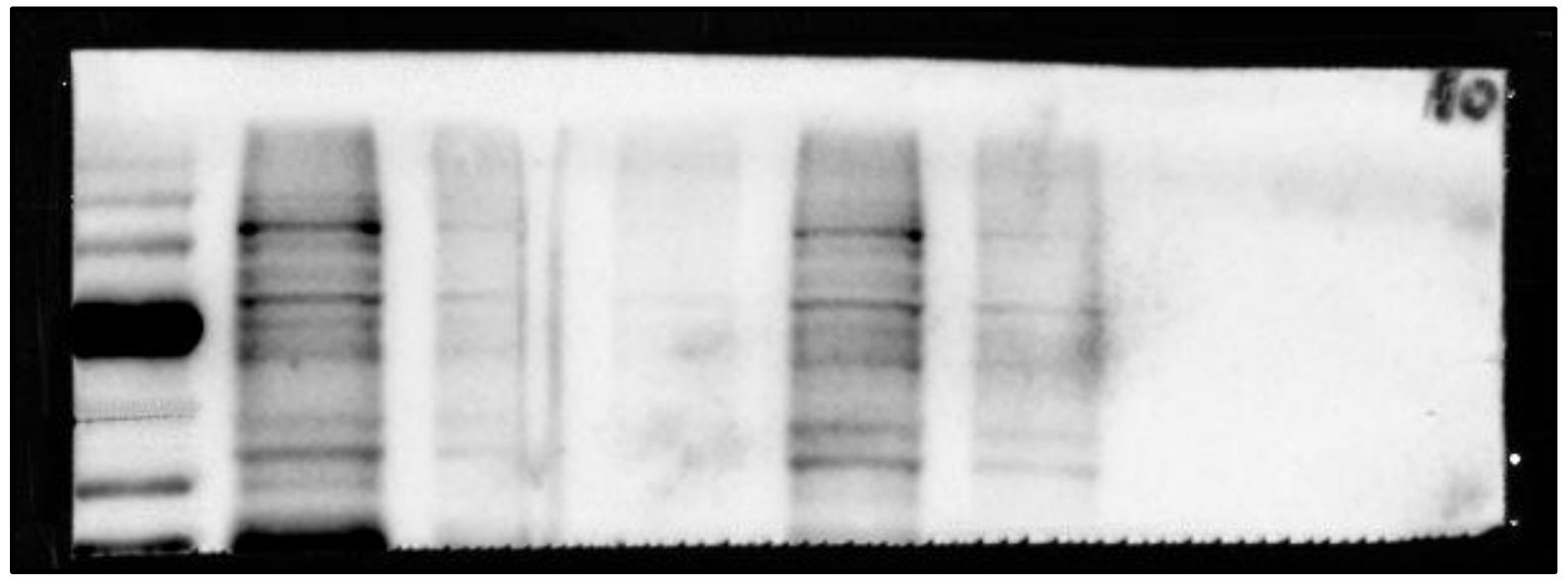

63kD

Parkin

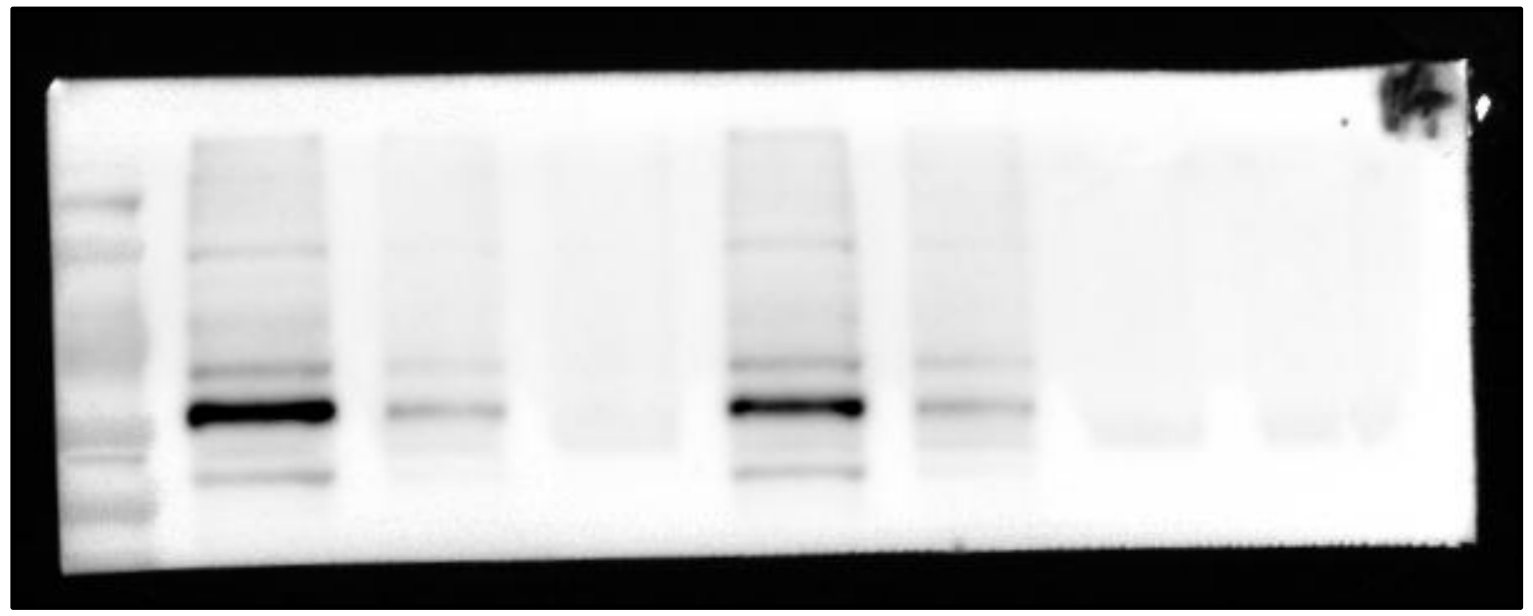

52kD

β-actin

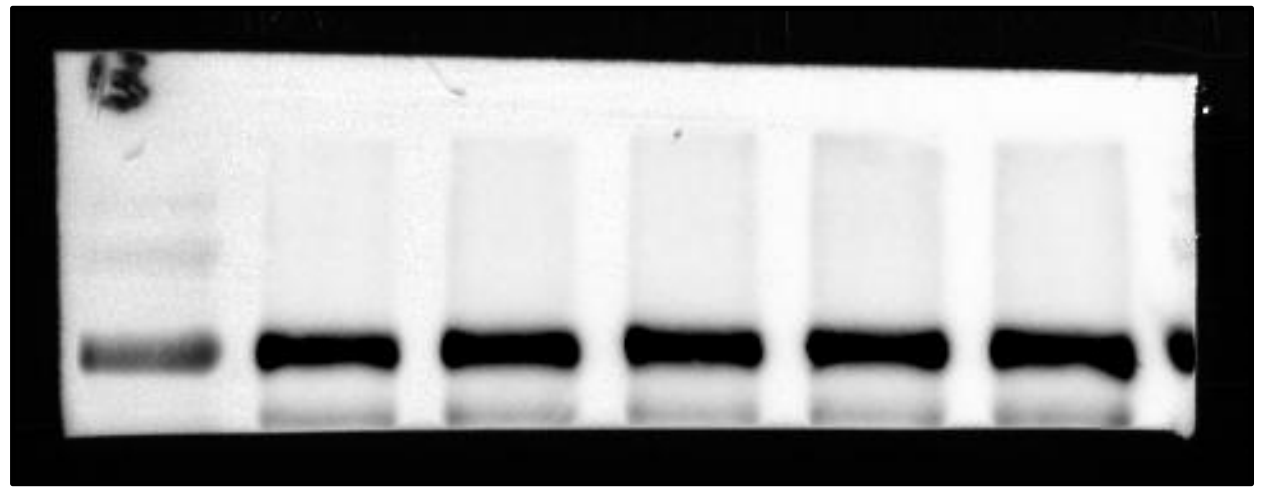

42kD

figure6 C

DRP1 83kD

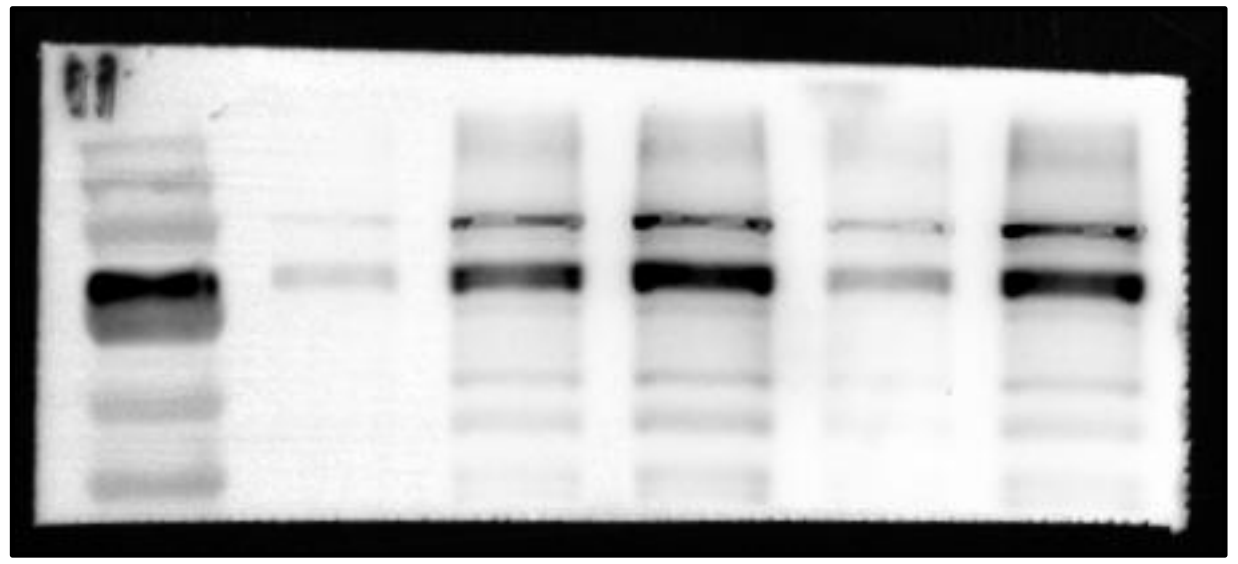

TOMM20 16kD

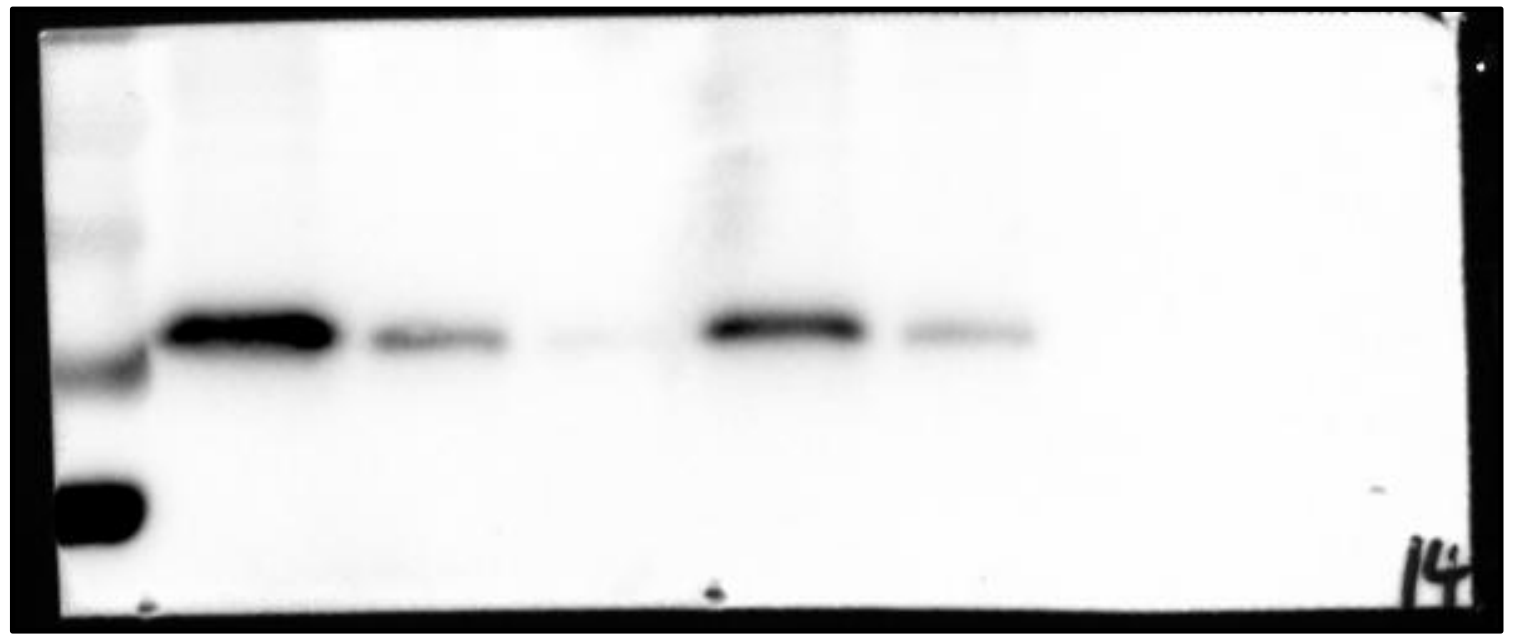

β-actin 42kD

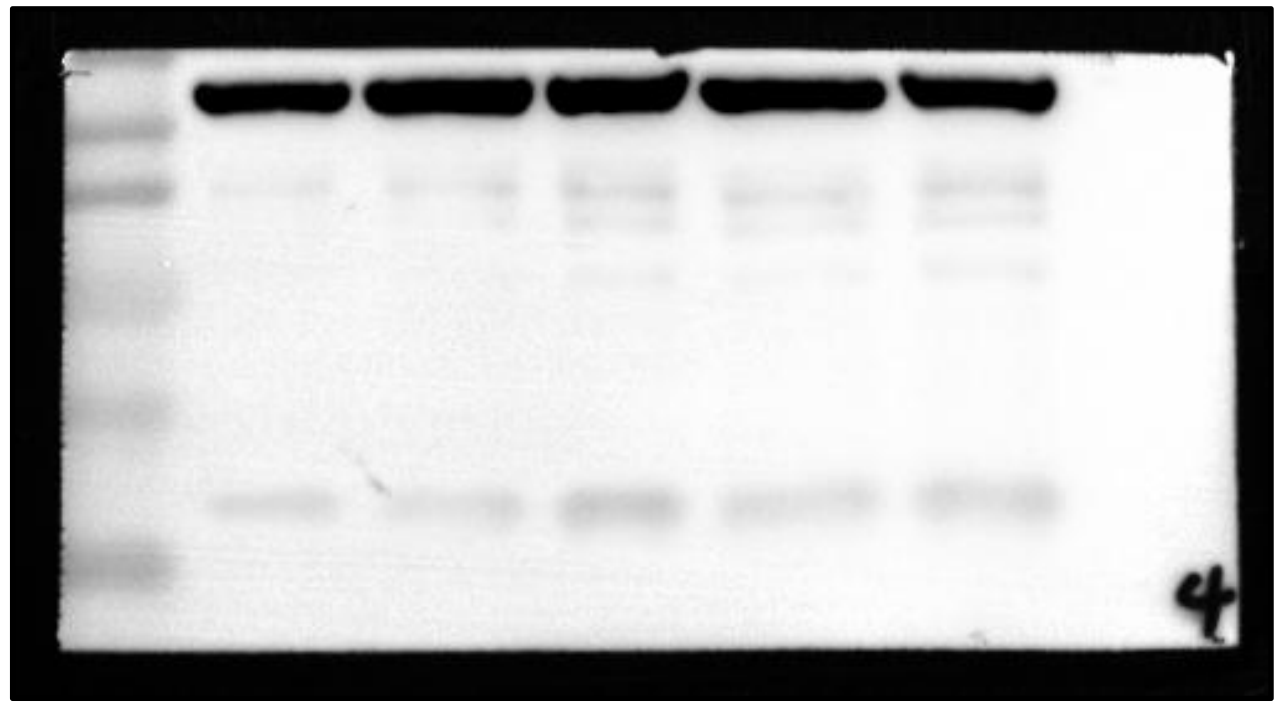

Supplementary Figure 7. Full-length western blot images corresponding to Figure 7.

Original full-length western blot images corresponding to the representative immunoblots presented in Figure 7. Molecular weight markers and all experimental lanes were retained.

figure7 B

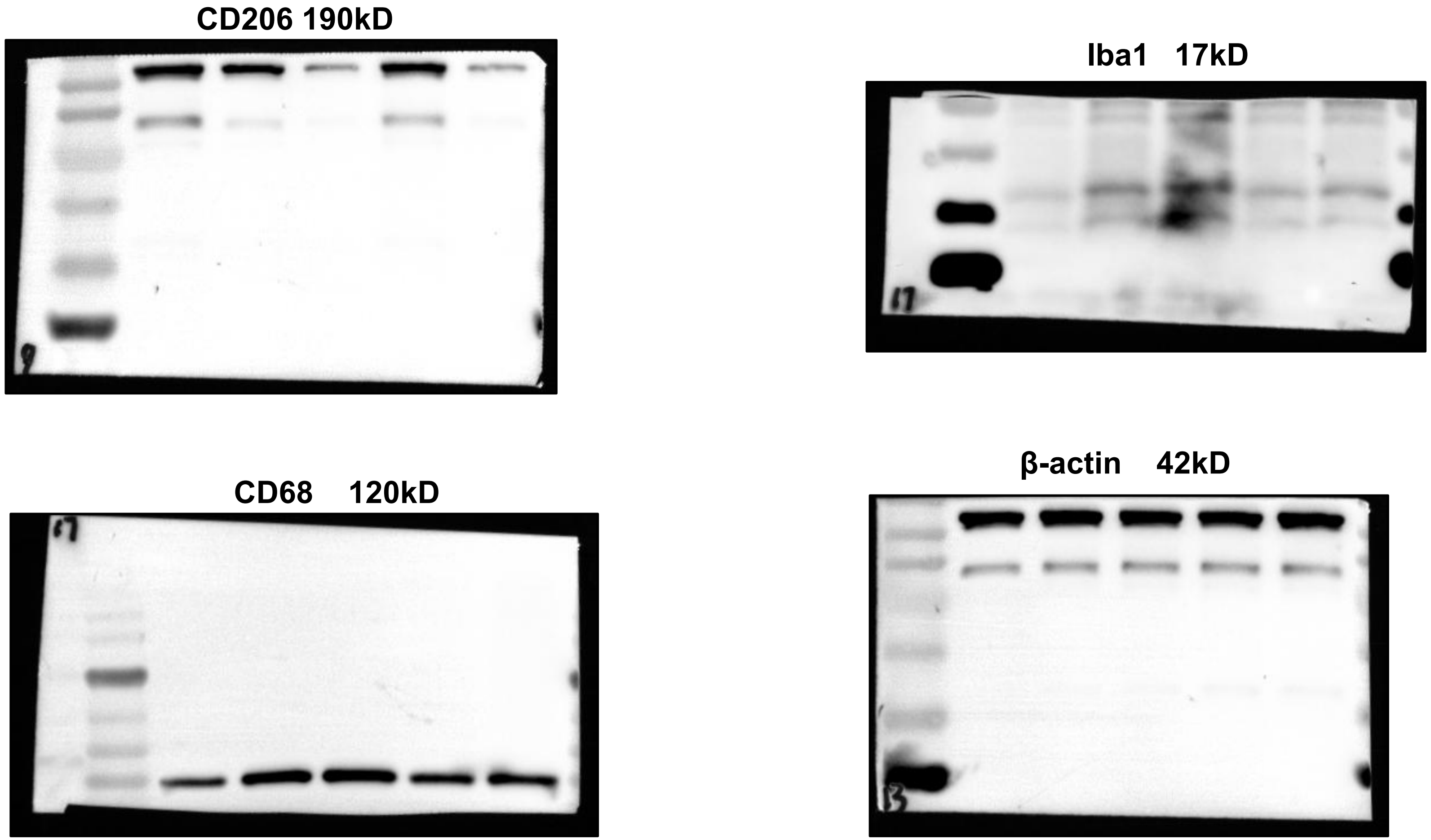

figure7 D

AMPK 64kD

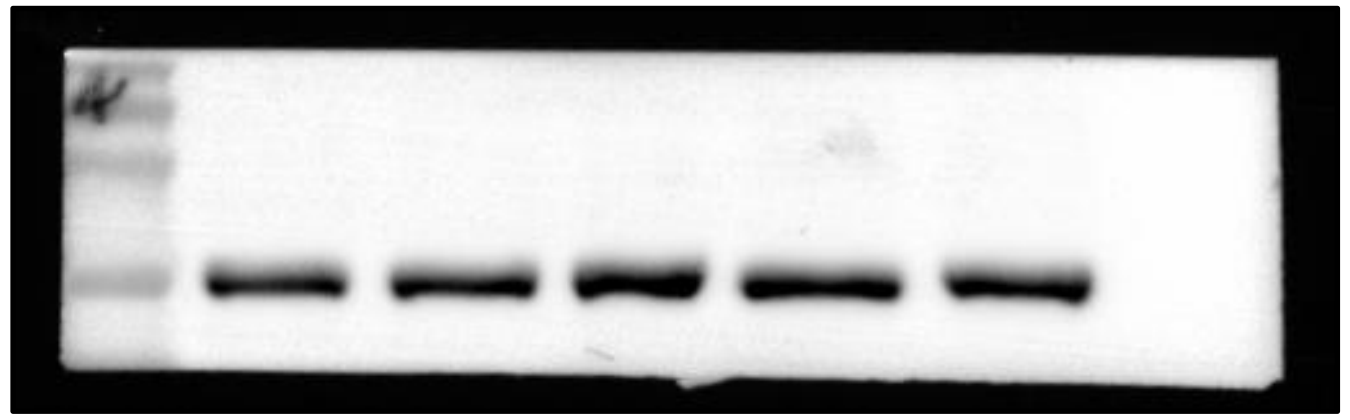

p-AMPK 62kD

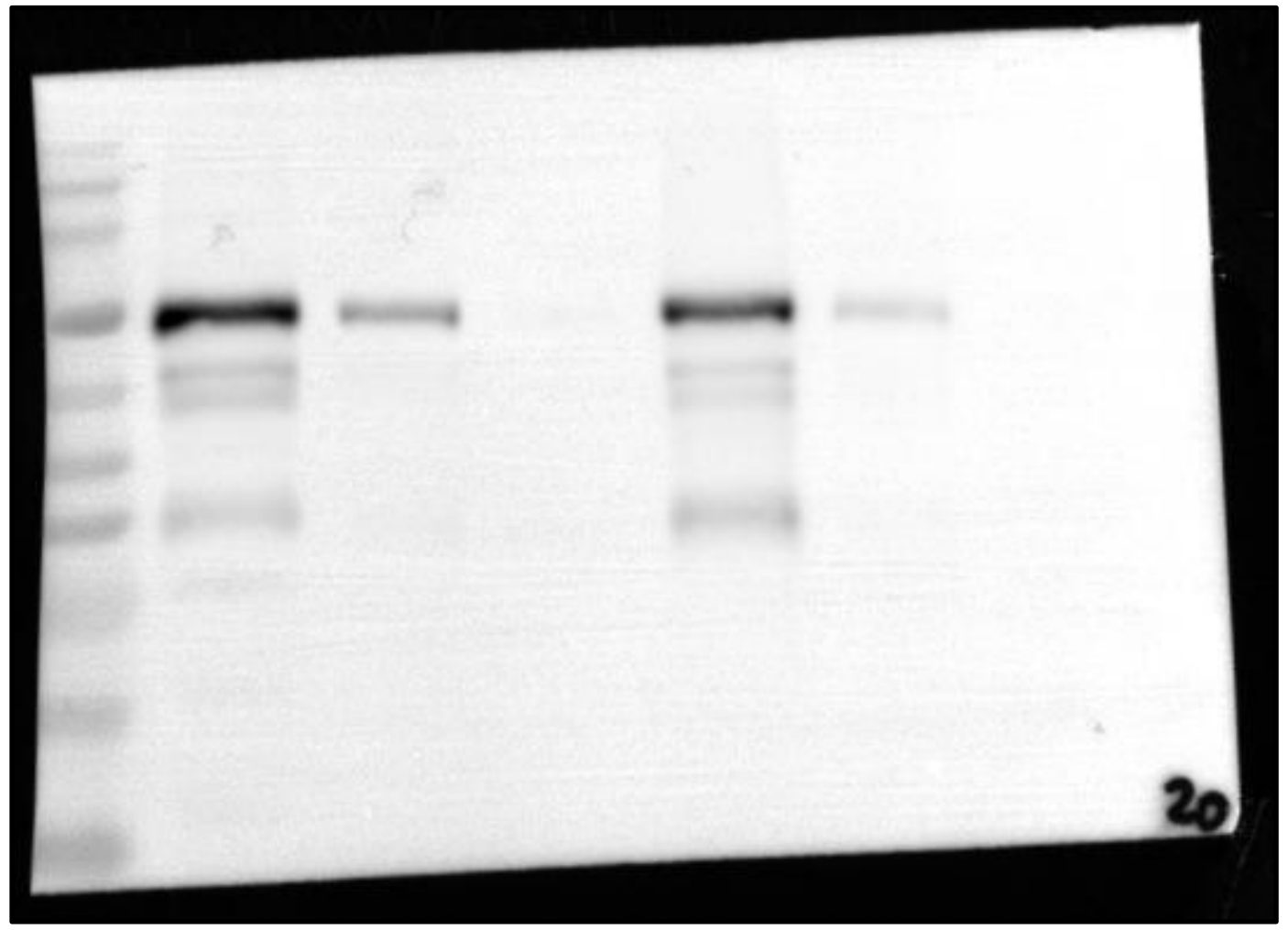

Pink1 63kD

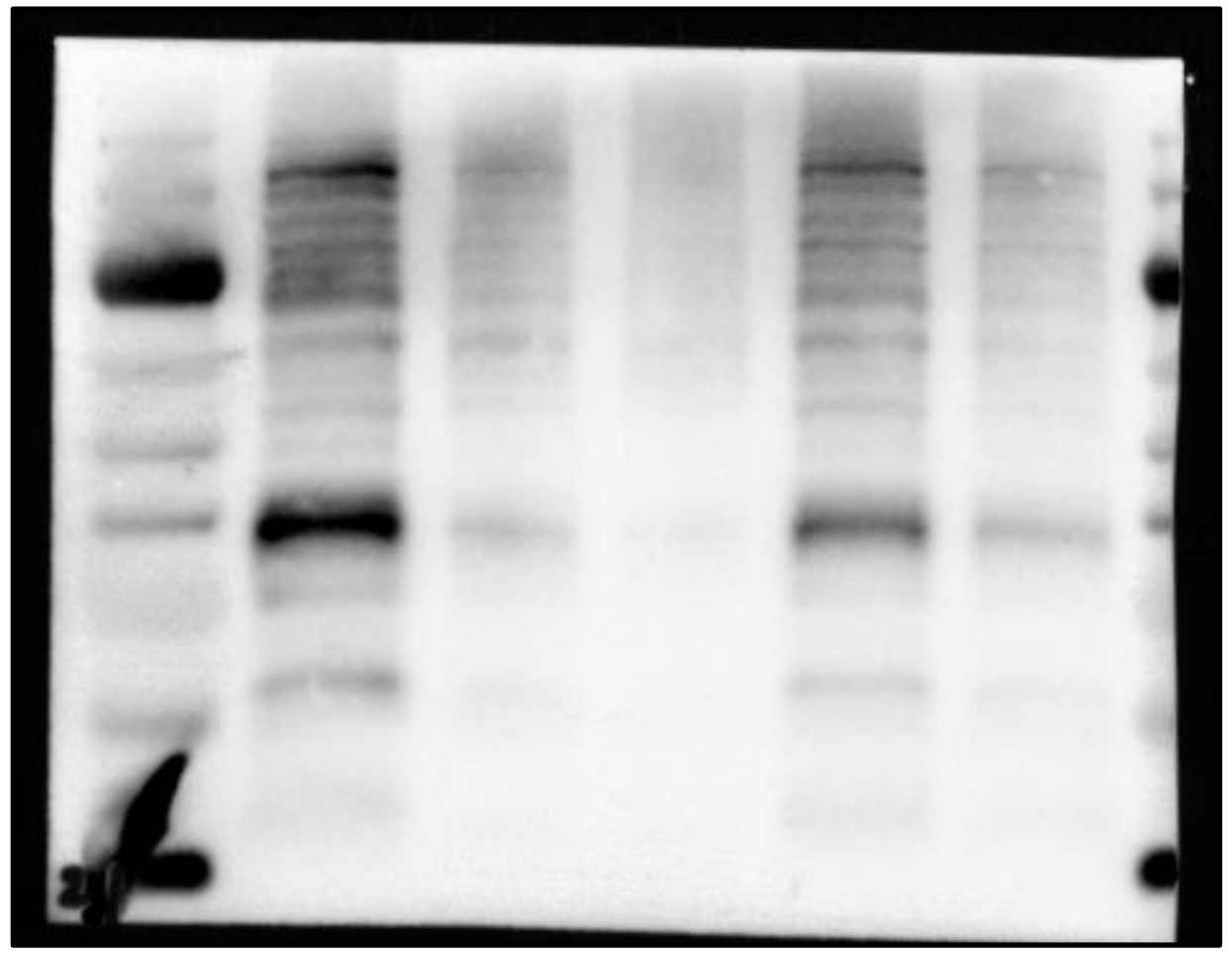

Parkin 52kD

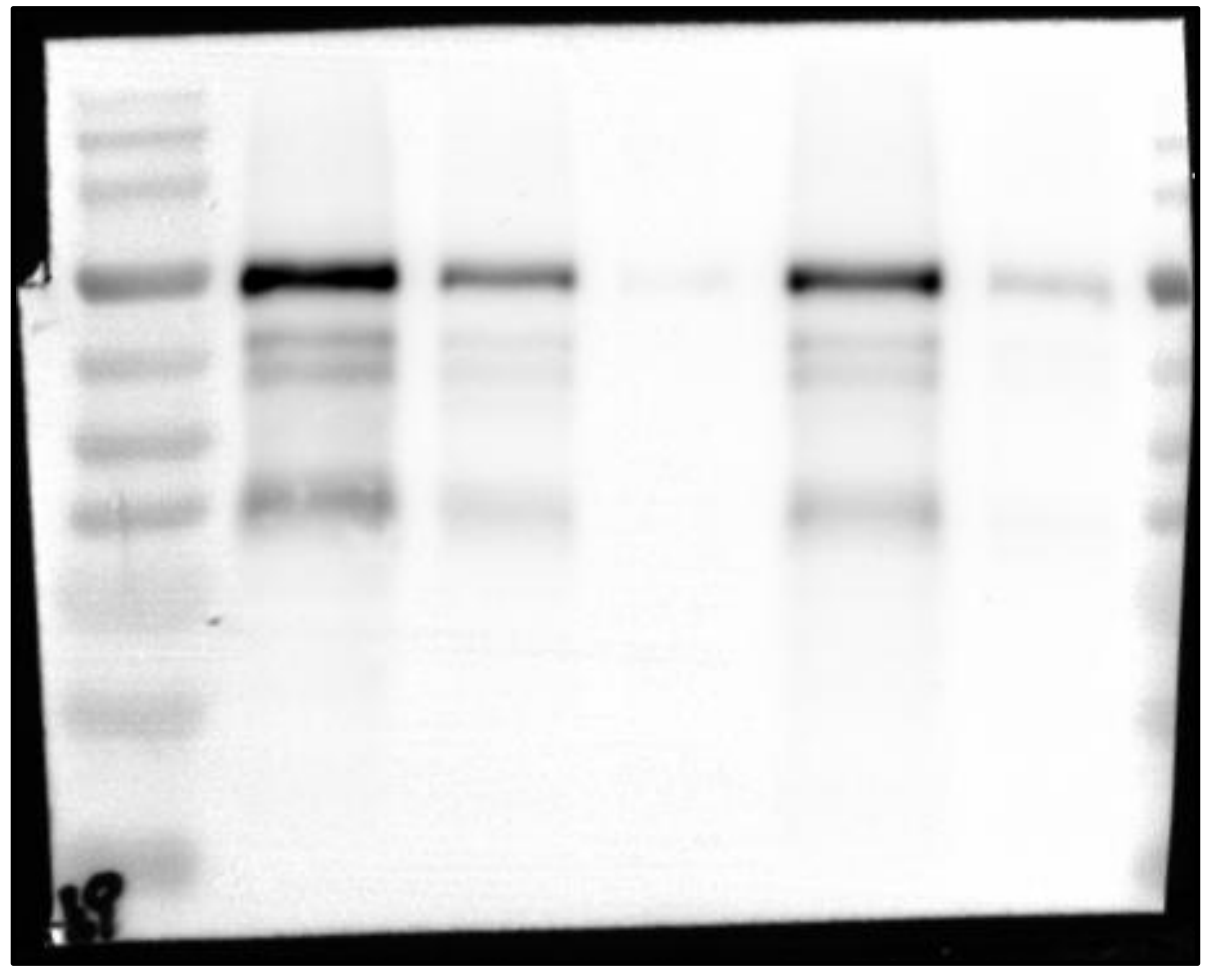

β-actin 42kD

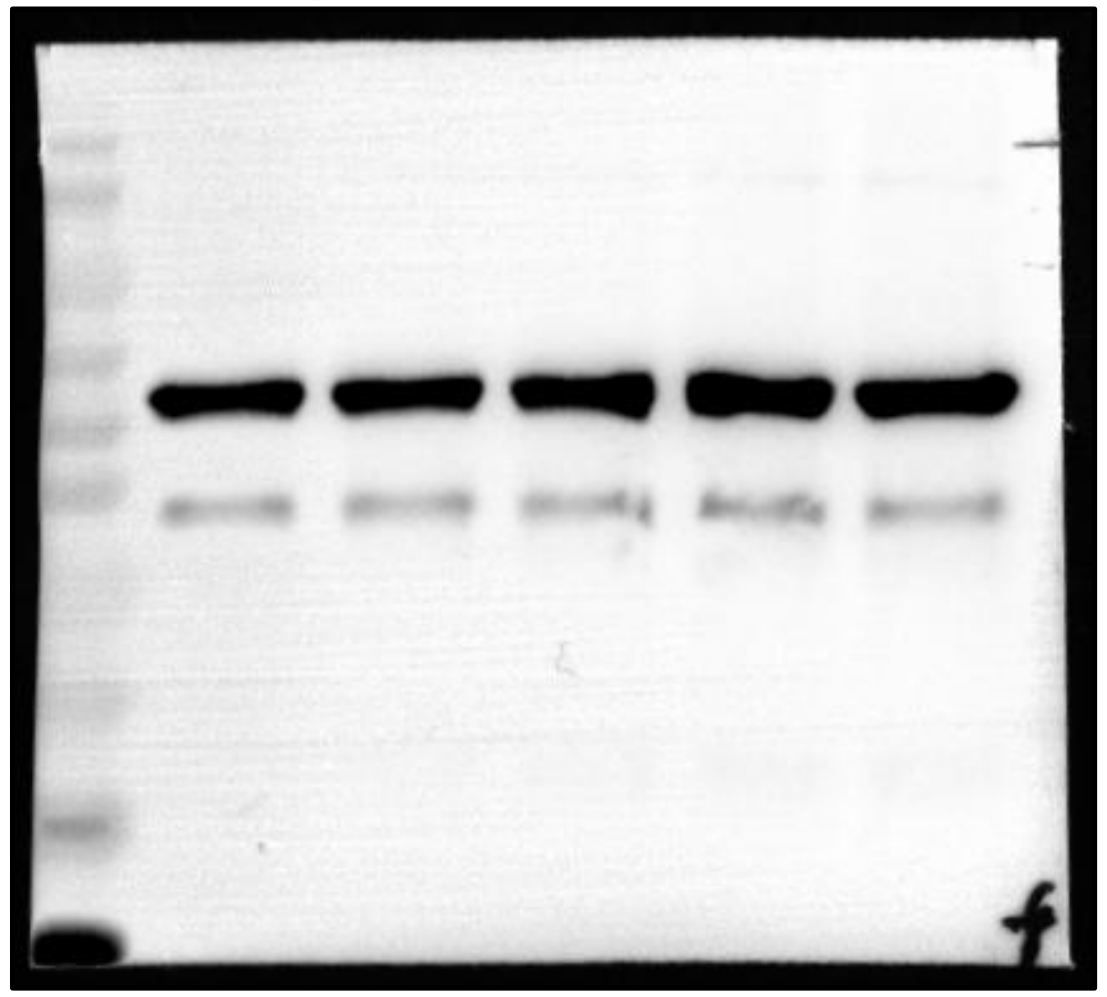

Supplement: Supplementary file 1 [file Data_Sheet_1.PDF]
